# A multiple reaction monitoring method for determining peanut (*Arachis hypogea*) allergens in serum using quadrupole and time-of-flight mass spectrometry

**DOI:** 10.1007/s00216-020-02508-9

**Published:** 2020-03-03

**Authors:** Charlotte M. Hands, Rebekah L. Sayers, Chiara Nitride, Lee A. Gethings, E. N. Clare Mills

**Affiliations:** 1 Division of Infection, Immunity and Respiratory Medicine, School of Biological Sciences, Manchester Institute of Biotechnology, Manchester Academic Health Sciences Centre, University of Manchester, Manchester, M1 7DN UK; 2 Waters Corporation, Stamford Avenue, Altrincham Road, Wilmslow, SK9 4AX UK

**Keywords:** Peanut allergen, Serum, Multiple reaction monitoring, Triple quadrupole, Time-of-flight mass spectrometry

## Abstract

Peanut is a major cause of severe IgE-mediated food allergic reactions, which can be exacerbated by factors, such as exercise, that may increase allergen uptake into the circulation. Enzyme-linked immunosorbent assays (ELISAs) have been used to determine allergen uptake into serum, but there are concerns over their specificity and a confirmatory method is required. Mass spectrometry (MS) methods have the potential to provide rigorous alternatives for allergen determination. A suite of peptide targets representing the major clinically relevant peanut allergens previously applied in food analysis were used to develop a targeted multiple reaction monitoring (MRM) method for determination of peanut in serum. Depletion of serum using affinity chromatography was found to be essential to allow detection of the peptide targets. A comparison of triple quadrupole and Q-TOF methods showed that one Ara h 2 peptide was only detected by the Q-TOF, the other peptide targets giving similar assay sensitivities with both MS platforms, although transitions for all the peptides were detected more consistently with the Q-TOF. The Q-TOF MRM assay detected peanut from spiked serum more effectively than the triple quadrupole assay, with Ara h 3 being detected down to 3 mg total peanut protein/L of serum, comparable with an Ara h 3–specific ELISA. The poor recoveries observed for both methods are likely due to loss of peanut immune complexes during the serum depletion process. Nevertheless, the Q-TOF MRM method has much promise to confirm the uptake of peanut proteins in serum samples providing immune complexes can be disrupted effectively prior to depletion.

**Electronic supplementary material:**

The online version of this article (10.1007/s00216-020-02508-9) contains supplementary material, which is available to authorized users.

**Figure:**
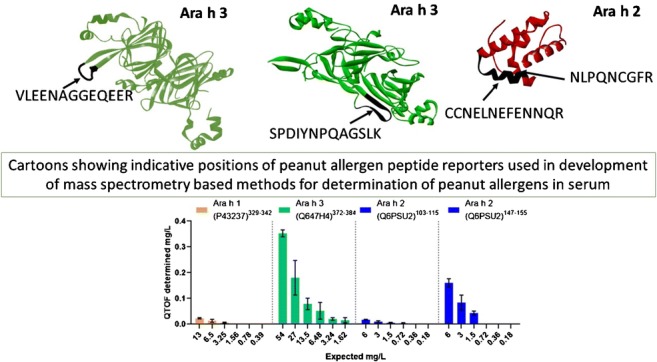
Graphical abstract

## Introduction

IgE-mediated food allergies are the result of an exaggerated immune reaction in response to innocuous environmental proteins termed allergens [1]. Such responses can occur to a variety of agents including dust, pollen, and foods, with the latter causing severe, even life-threatening reactions in some individuals. Food allergy affects up to around 4% of adults across Europe although rates vary widely, as do the causative foods [2]. For example, rates of allergy to peanut is more important in countries, such as the UK, where it is estimated to cause food allergy in around 2% of school age children [3]. Peanut has also been shown to be a significant cause of anaphylaxis in a number of countries [4–6]. Around 13 allergenic peanut proteins have been identified which include the major clinically relevant allergens, the cupin seed storage protein allergens known as Ara h 1 and Ara h 3 and the 2S albumin members of the prolamin superfamily, Ara h 2 and 6 [7, 8]. It has been proposed that factors, such as resistance to digestion, may allow sufficient protein to be presented to the immune system to allow allergens to both sensitise and elicit allergic reactions [9, 10]. Indeed, there is evidence that peanut allergens can be taken up into the circulation, with exercise apparently increasing uptake in healthy volunteers. Peanut and gluten can be detected in the bloodstream after ingestion using ELISA-based methods, in both allergic and non-allergic individuals [11–14].

However, such methodology has some limitations, such as antibody cross-reactivity with closely related proteins, leading to false positive results [15]. An alternative method, which could provide sequence-level confirmation of the presence of food allergens in the circulation, is targeted liquid chromatography mass spectrometry (LC-MS). Target peptides have previously been identified for major peanut allergens and used to develop LC-MS methods for detection of peanut in food [16–21], a set of which were used to develop a triple quadrupole multiple reaction monitoring (QQQ-MRM) method which had a greater sensitivity when a microfluidic separation was used for analysis of a chocolate-containing dessert matrix [22]. This method has now been adapted to the analysis of peanut allergen peptide targets in serum and compared with a quadrupole-time-of-flight (Q-TOF) MS approach for analysis of biomarkers in undepleted plasma [23], which had a superior sensitivity when analysing tissues [24]. The ion mobility separation together with greater mass accuracy and resolution provided by the TOF allows better discrimination of the target ion from the serum-derived background ions [25–27].

## Materials and methods

### Materials

All reagents and chemicals were of analytical grade unless otherwise stated. Blank human serum, iodoacetamide, dithiothreitol (DTT), ammonium bicarbonate, and Glu-fibrinopeptide were purchased from Sigma-Aldrich (Dorset, UK). MS-grade trypsin Gold was purchased from Promega (WI, USA). RapiGest^™^ (sodium 3-[(2-methyl-2-undecyl-1,3-dioxolan-4-yl)methoxy]-1-propanesulphonate) was from Waters Corporation (MA, USA). Multiple Affinity Removal LC Column Human 6 and associated buffers, spin filters, and concentrators were purchased from Agilent Technologies (Cheshire, UK). Ara h 1, 2, 3, and 6 ELISA kits were purchased from Indoor Biotechnologies (Cardiff, UK). Protein electrophoresis buffers and reagents were from Thermo Fisher and comprised the following: SimplyBlue SafeStain Coomassie^®^ G-250 protein stain, Mark 12TM Unstained standard, NuPAGE Bis-Tris 4–12% acrylamide precast gels, lithium dodecyl sulphate (LDS) sample buffer (4× concentrate), and 2-(*N*-morpholino)ethanesulphonic acid (MES) buffer (20× concentrate). AQUA peptides that were synthesised with a heavy isotope-labelled 13C(6)15N(4) C-term arginine or 13C15N(2) C-term lysine were purchased from JPT Peptide Technologies (Berlin, Germany) and supplied as the lyophilised trifluoroacetate salt. Synthetic peptide targets were as follows: Ara h 1 (P43237)^329–342^ (VLLEENAGGEQEER), Ara h 1 (P43237)^555–577^ (DLAFPGSGEQVEK), Ara h 3 (Q647H4)^372–384^ (SPDIYNPQAGSLK), Ara h 3 (Q647H4)^25–41^ (QQPEENACQFQR), Ara h 2 (Q6PSU2)^103–115^ (CCNELNEFENNQR), Ara h 2 (Q6PSU2)^147–155^ (NLPQQCGLR), Ara h 6 (Q647G9)^136–144^ (CDLDVSGGR), Ara h 7 (B4X1D4)^143–151^ (NLPQNCGFR). All cysteine residues in the peptide sequences are modified by means of carbamidomethylation (C[+ 57 Da]). All peptides were reconstituted in 10 μL of 0.1% (v/v) formic acid and 2% (v/v) acetonitrile in HPLC-grade water and were stored at − 80 °C prior to use. Two batches of isotopically labelled peptides were utilised in the experimental work (see Electronic Supplementary Material (ESM) Table S1.4). Set 1 comprised a suite of eight synthetic peptides used for the initial development of the QQQ method. Peptides were produced with TFA as a counter ion, target mass confirmed by LC-MS, and purity confirmed as > 95% by HPLC trace at 220 nm. Set 2 comprised a subset of four synthetic peptides (Ara h 1 (P43237)^329–342^, Ara h 3 (Q647H4)^372–384^, Ara h 2 (Q6PSU2)^103–115^, Ara h 2 (Q6PSU2)^147–155^), the concentrations of which were determined by amino acid analysis. These were used for the Q-TOF analysis.

### Peanut protein spiking of serum

Peanut protein extracts were prepared from raw peanuts (var. Runner) obtained from PepsiCo (Leicester, UK) using 50 mM TRIS-HCl, pH 8.8, and the protein content determined using a 2D Quant-Kit^™^ (GE Healthcare Life Sciences, Buckinghamshire, UK), with bovine serum albumin as a standard, as previously described [16]. The extract was analysed by SDS-PAGE using the Nu-PAGE gel system (Invitrogen, Paisley, UK) with 4–12% Bis-Tris precast gradient gels, and proteins were separated at 200 V for 35 min [16]. Gels were stained using Simply Blue Safe Stain (Invitrogen, Paisley, UK) and visualised using a Typhoon TRIO variable mode imager (GE Healthcare, Buckinghamshire, UK). Densitometry was performed using the ImageQuant Software (GE Healthcare, Buckinghamshire, UK). The raw peanut protein extract (1 mg/mL protein) was added to the human serum sample to create a solution of 100 mg peanut protein per litre of serum. This was used to create a serial freshly prepared spiked dilution series from 3 to 100 mg/L total peanut protein using further aliquots of blank serum. Each set of spiked serum samples was freshly prepared prior to depletion.

### Serum sample preparation

Serum samples were depleted of high-abundance serum proteins (such as albumin, IgG, IgA, transferrin, fibrinogen, antitrypsin, and haptoglobin) using either a ProteoPrep^®^ 20 Plasma Immunodepletion Kit (Sigma, UK) or a Multiple Affinity Removal System Hu-6 (human; MARS Hu-6) (Agilent, UK) 4.6 × 5 mm column attached to a Shimadzu Prominence UFLC system (Shimadzu, Kyoto, Japan). Depletion was performed using buffers provided with the kit according to the manufacturer’s instructions. Prior to reduction and alkylation, serum (20 μL) was diluted by addition of 100 μL of the ProteoPrep kit equilibration buffer to be of equivalent to depleted samples. Samples were prepared for mass spectrometry using two different methods:

1. In-solution digestion: Serum samples, before and after depletion, were then further diluted into 50 mM ammonium bicarbonate containing 0.1% (w/v) RapiGest^™^ and heated at 80 °C for 45 min. Samples were then reduced by the addition of DTT to 5 mM, heated at 60 °C for 30 min, and clarified by centrifugation. Alkylation was performed by the addition of iodoacetamide to 10 mM and incubating samples in the dark at ambient conditions for 30 min. Trypsin digestion was performed using ~ 1:20 (w/w) trypsin:protein ratio. The total serum protein concentration was estimated to be 70 mg/mL based upon literature review, taking the mid-point of an agreed serum protein reference intervals [28]. Trypsin was prepared by diluting a 1-mg/mL stock solution in 50 mM acetic acid to 0.1 mg/mL in 50 mM ammonium bicarbonate; 40 μL of this was added to each serum sample and incubated at 37 °C for 16 h. Digestion was quenched via the addition of trifluoroacetic acid (TFA) to 1% (v/v) before clarifying samples by centrifugation at 13,000*g* for 25 min. The supernatants were removed and stored at − 20 °C until required.
2. Filter-aided digestion: A method previously developed for analysis of peanut in food was adapted for analysis of serum [22]. It employed a 3-kDa molecular weight cutoff centrifugal filter (Amicon micro, VWR International, Leicester, UK) using the in-solution digestion conditions described above except a second addition of trypsin was made after the first 3 h of digestion. The centrifugation steps allowed the recovery and concentration of the digested peptides in the filtrate. Formic acid was then added to a final concentration of ~ 0.1% (v/v) to hydrolyse the acid-labile detergent and inactivate any remaining trypsin, filters centrifuged at 15,000×*g* for 20 min, and the peptide-containing filtrate collected. Formic acid was then added to a final concentration of 1% (v/v) prior to analysis.

Serial isotopic dilutions (SIDs) of AQUA peptides were then prepared from 0 to 5 fmol of heavy labelled peptide per microlitre of processed serum sample. For peanut protein–spiked serum samples, a 5-nM isotopically labelled peptide spike solution was added to each reduced, alkylated, and digested serum sample to give a final concentration of 5 fmol/μL.

### 1D polyacrylamide gel electrophoresis

Raw peanut extracts were analysed by 1D PAGE using the Nu-PAGE gel system (Invitrogen, Paisley, UK). LDS sample buffer was added (1:4 sample to buffer ratio (v/v)), and samples (1 mg protein/mL) were heated for 5 min at 70 °C. Samples (10 μL per well) and molecular weight markers (Mark-12, Invitrogen, Paisley, UK) were loaded onto 4–12% Bis-Tris precast gradient gels, and proteins were separated at 200 V for 35 min. Gels were stained using Simply Blue Safe Stain (Invitrogen, Paisley, UK) and visualised using a Typhoon TRIO variable mode imager (GE Healthcare, Buckinghamshire, UK). Densitometry was performed using the ImageQuant Software (GE Healthcare, Buckinghamshire, UK) to determine relative abundance of individual allergens.

### ELISA

Ara h 1, 2, 3, and 6 were quantified in peanut extracts and spiked depleted serum samples using allergen-specific sandwich ELISAs. Assays were performed according to the manufacturer’s instruction, which are summarised as follows: Polystyrene NUNC MaxiSorp 96-well plates were coated overnight at 4 °C with 100 μL/well of the appropriate antibody in 50 mN carbonate-bicarbonate buffer, pH 9.6. After washing three times with phosphate-buffered saline (PBS; 137 mM NaCl, 1.5 mM KH_2_PO_4_, 8 mM Na_2_HPO_4_, 8 mM Na_2_HPO_4_, 2.7 mM KCl) containing 0.05% (v/v) Tween-20 (PBS-T), plates were incubated with 200 μL/well of 1% (w/v) bovine serum albumin (BSA) in PBS for 2 h under ambient conditions. Plates were then washed a further three times with PBS-T, and either 100 μL/well in triplicate of the appropriate peanut allergen standards ranging from 125 to 0 ng/mL, or 100 μL/well in triplicate of each depleted serum sample was applied to the plate and incubated for 1 h under ambient conditions. After washing a further three times with PBS-T, 100 μL/well of biotinylated antipeanut allergen mAB (diluted 1:1000 (v/v) in 1% (w/v) BSA in PBS-T) was added to the plate and incubated for 1 h under ambient conditions. The plate was then washed three times with PBS-T before adding 1 μL/well of streptavidin-peroxidase (diluted 1:1000, (v/v) in 1% (w/v) BSA-PBS-T) and incubated for 30 min under ambient conditions. Plates were washed a further three times with PBS-T before the addition of 100 μL/well of 1 mM 2,2′-azino-bis(3-ethylbenzothiazoline-6-sulphonic acid) (ABTS) in 70 mM citrate-phosphate buffer, pH 4.2, containing 0.1% (v/v) H_2_0_2_. The absorbance of each well was read at 405 nm.

### Mass spectrometry analysis

A Skyline method was derived using the target peptide sequences with fixed modifications for the custom-synthesised AQUA peptides being the isotopically labelled 13C(6)15N(4) C-term arginine or 13C15N(2) C-term lysine and the carbamidomethylation of the cysteine. For each target peptide, three MRM transitions were selected corresponding to precursor ions with a 2^+^ charge paired with the resulting y-fragment ions and peptide transitions exported for use with the Xevo^®^ TQ-S mass spectrometer (Waters, Wilmslow, UK) (ESM Table S1.3). Triplicate injections of each sample were performed for the serial isotopic dilutions, whilst three biological replicates of the peanut protein–spiked serum were prepared and each analysed with three technical replicates. Target peptides were analysed using the two different types of mass spectrometry as follows.

### QQQ analysis

Peptides were analysed using Xevo^®^ TQ-S (Waters, Wilmslow, UK) operated in positive ion MRM mode, which was coupled to an Waters ACQUITY M-Class UPLC system (Waters, Milford, MA, USA) equipped with an iKey source and iKey peptide BEH C18, 130 Å, 1.7 μm, 150 μm × 50 mm (Waters, Milford, MA, USA). The iKey was heated to 35 °C whilst samples were maintained at 8 °C prior to analysis. Triplicate injections using a sample volume of 3 μL were employed. Separation was performed using a flow rate of 1.2 μL/min and the system initially equilibrated in 98% HLPC-grade water containing 1% (v/v) acetonitrile and 0.1% (v/v) formic acid (buffer A) and 2% (v/v) of 98% acetonitrile containing 1% (v/v) HLPC-grade water and 0.1% (v/v) formic acid (buffer B). The gradient was then ramped from 2 to 45% buffer B over 10 min and then to 80% (v/v) over a further 2 min to rinse column before re-equilibrating at 2% buffer B. The optimised source parameters used were as follows: capillary voltage 3.5 kV; cone voltage 30 V; source temperature 150 °C; cone gas flow 150 L h^−1^; collision gas flow 0.13 mL min^−1^. Argon was used as the collision gas. For the quadrupole and the collision cell, the parameters were as follows: LM resolution, 3.2; HM resolution, 14.2; ion energy, 0.3 V. The mass spectrometer was operated with a spectral acquisition time of 15 min and each sample was analysed in triplicate. Targeted analysis was performed using the list of predetermined transitions (ESM Table S1.3).

### Q-TOF analysis

Peptides were analysed on a Waters ACQUITY M-Class Ultra Performance LC (Waters, Wilmslow, UK) interfaced to a Synapt G2-Si QTOF mass spectrometer (Waters, Milford, MA, USA) operating in positive ion TOF-MRM mode. During data acquisition, a low collision-induced dissociation (CID) energy, at 4 eV, was applied across the transfer ion guide using argon as the CID gas. During high CID energy, a ramp of 24–45 eV was applied. The cone voltage was set as 35 V with a scan time of 0.5 s. LockSpray of Glu-fibrinopeptide (GFP) *m*/*z* at 785.8426 was used to maintain mass accuracy throughout the chromatographic run. Samples (3 μL in triplicate) were loaded onto an analytical HSS T3 C18 75 μm × 150 mm, 1.7-μm analytical column (Waters, Milford, MA, USA) and eluted over a linear gradient at a flow rate of 3 μL/min. Peptides were kept at 8 °C prior to analysis. Targeted analysis was performed using the list of predetermined transitions (see “Data analysis”). The optimised source parameters used were as follows: capillary voltage 3.2 kV; cone voltage 35 V; source temperature 100 °C; cone gas flow 35 L h^−1^, extraction cone, 3.0 kV; desolvation temperature, 250 °C; cone flow, 35 L/h; desolvation flow, 800 L/h. For the quadrupole and the collision cell, the parameters were as follows: LM resolution, 5.0; HM resolution, 15.0; ion energy, 0.2 V; prefilter, 2.0 V; gas flow 0.10 mL/min, with a scan time of 0.5 s. Argon was used as the collision gas. In addition to targeted MS/MS, a full scan profile was also acquired over the mass range of 50–2000 Da during the course of the gradient. Targeted analysis was performed using the list of predetermined transitions (ESM Table S1.3). The transitions utilised for the QQQ and Q-TOF experiments were the same as were the optimised collision energy profiles since the same collision cell is used in both instruments.

### Data analysis

Data files were imported into Skyline [29] and visually inspected to check the retention times and peak areas and then evaluated for reproducibility as described in the ESM Section S1.1 and Tables S1.2, S1.3, and S1.5. The total peak intensity data for the SID series were then imported into GraphPad Prism version 7.01 for Windows (GraphPad Software, San Diego, CA, USA, www.graphpad.com), and a linear regression fitted using least squares. The limit of detection (LOD) of the instrument was taken as the lowest peptide concentration at which the peptide transition could be differentiated from the noise [30] and was calculated using the calibration curve, which tends to over-estimate the LOD [30] as follows:

$$ {\displaystyle \begin{array}{c}(1)\ Y=\frac{3\times \mathrm{Standard}\ \mathrm{deviation}\ \mathrm{of}\ \mathrm{the}\ \mathrm{residuals}\ \left(\mathrm{sy}|x\right)}{\mathrm{Slope}+\mathrm{the}\ \mathrm{average}\ \mathrm{lowest}\ \mathrm{true}\ \mathrm{intensity}\ \mathrm{from}\ \mathrm{the}\ \mathrm{linear}\ \mathrm{portion}\ \mathrm{of}\ \mathrm{the}\ \mathrm{curve}}\\ {}(2)\ X=\frac{\left(Y-y\ \mathrm{intercept}\right)}{\mathrm{slope}.}\end{array}} $$

The LOD was then calculated by taking the inverse of *X*.

The lower limit of quantification (LLOQ) was the lowest concentration of peptide at which quantitative measurements calculated could be made [31] and was defined as

$$ LLOQ=3\times LOD. $$

The peak area ratios of the endogenous light peanut peptide reporter to the corresponding heavy labelled peptide standard were calculated based on total ion intensity of all three MRM transitions for each target peptide. The ratio was then multiplied by the concentration of the heavy spike to infer the peptide concentration in the unknown sample, taking into account the dilution during sample preparation. The peptide concentration was then converted to peanut allergen protein using a set of standard molecular weights for the mature allergens from which the peptides were derived [22, 32] (ESM Table S1.4). The data analysis for the ELISA was undertaken using the standard curve data generated using allergen standards supplied with each kit, and the concentration of the unknown samples interpolated using GraphPad Prism. Descriptive statistical analysis was also undertaken using GraphPad Prism.

## Results and discussion

### Effect of sample preparation for analysis of peanut peptide targets in serum

A set of eight peanut allergen peptide markers, previously used for development of a multiple reaction monitoring (MRM) method for analysis of peanut in food [22], were evaluated for use in a MS-based method for confirmation of peanut allergens in serum. They represent the major clinically relevant peanut allergens belonging to the cupin superfamily, Ara h 1 and Ara h 3, together with several members of the prolamin superfamily of allergen, the 2S albumins known as Ara h 2, 6, and 7.

Initially, the effect of different sample preparation workflows was investigated using a QQQ mass spectrometer working in MRM mode monitoring the heavy labelled peptides (ESM Fig. S1.1). In stage 1 of the method development, a HPLC column and a spin cartridge were evaluated for the depletion, comparing them to non-depleted serum. To this extent, the background serum proteome was digested using a canonical in-solution digestion protocol. Once the best performing condition for the depletion was optimised, in stage 2, we compared the efficiency of the canonical in-solution digestion with a filter-aided digestion protocol. Another approach used to improve assay sensitivity through the use of solid-phase extractions cartridges when analysing allergens in foods [33] was also explored but gave no benefit in enhancing assay sensitivity (data not shown). The effect of the different sample preparation workflows on the detection of individual heavy labelled peptide targets, is described below. The ion chromatograms of monitored heavy labelled peptides using QQQ and Q-TOF MRM are shown in ESM Fig. S2.1.

#### Ara h 1

**Fig. 1:**
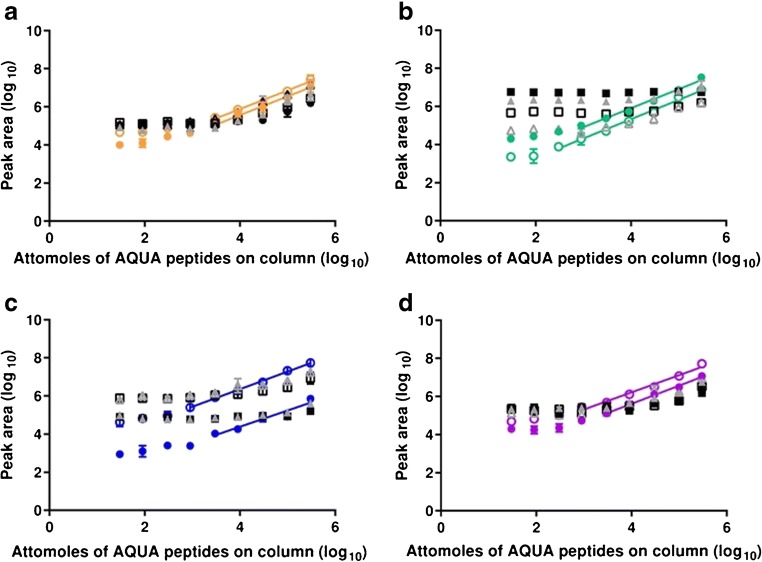
Serial isotopic dilution curves and corresponding transitions for heavy labelled peanut allergen peptide markers in depleted serum analysed using the QQQ-MRM method. Peptides were as follows: **a** Ara h 1 (P43237)^329–342^ (VLLEENAGGEQEER; solid symbols) and Ara h 1 (P43237)^555–577^ (DLAFPGSGEQVEK; open symbols); **b** Ara h 3 (Q647H4)^25–41^ (QQPEENACQFQR; solid symbols) and Ara h 3 (Q647H4)^372–384^ (SPDIYNPQAGSLK; open symbols); **c** Ara h 2 (Q6PSU2)^103–115^ (CCNELNEFENNQR; solid symbols) and Ara h 2 (Q6PSU2)^147–155^(NLPQQCGLR; open symbols); **d** Ara h 6 peptide Ara h 6 (Q647G9)^136–144^ (CDLDVSGGR; solid symbols) and Ara h 7 (B4X1D4)^143–151^ (NLPQNCGFR; open symbols). Peptides were diluted in either undepleted serum (black symbols and lines), serum depleted using a ProteoPrep spin column (grey symbols and lines) or serum depleted with a MARS column (coloured symbols and lines). Calibration curves were created using peptide concentrations of 0–100 fmol/μL and using samples with qualifying signal-to-noise ratios and ratios of quantifying peak area to total peak area

**Fig. 2:**
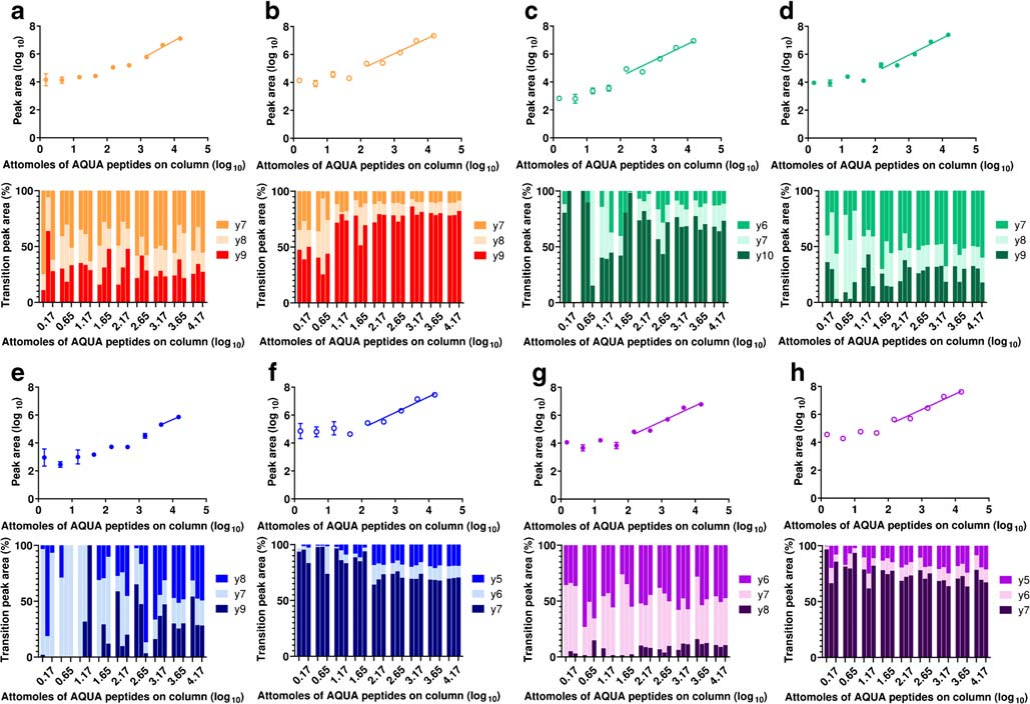
Serial isotopic dilution curves and corresponding transitions for heavy labelled peanut allergen peptide markers in depleted serum analysed using the QQQ-MRM method. Peptides were as follows: **a** Ara h 1 (P43237)^329–342^; **b** Arah1 (P43237)^555–577^; **c** Arah3 (Q647H4)^25–41^; **d** Arah3 (Q647H4)^372–384^; **e** Ara h 2 (Q6PSU2)^103–115^; **f** Ara h 2 (Q6PSU2)^147–155^; **g** Arah6 (Q647G9)^136–144^; **h** Arah7 (B4X1D4)^143–151^. Serum samples were depleted with a MARS-Hu6 column. Calibration curves were created using peptide concentrations from 0 to 15 fmol/μL using samples with qualifying signal-to-noise ratios and ratios of quantifying peak area to total peak area

Peptides Ara h 1 (P43237)^329–34^ and Ara h 1 (P43237)^555–577^ showed a similar behaviour with neither peptide being detected in serum (ESM Fig. S2.2), and only Ara h 1 (P43237)^555–577^ being detected in serum depleted using the ProteoPrep spin column (Table 1, Fig. 1a, and ESM Fig. S2.3). Both peptides could be detected in serum depleted using the MARS Hu-6 column, being quantified at levels ranging from 8.43 (Ara h 1 (P43237)^329–34^) to 22.72 (Ara h 1 (P43237)^555–577^) fmol of peptide on-column (Table 1, Fig. 1a, and ESM Fig. S2.4). The peptides showed a similar fragmentation pattern to that previously described with the y 7, 8, and 9 transitions being the best performing [16, 22] (ESM Fig. S2.5). The transitions for the Ara h 1 (P43237)^555–577^ peptide were again unevenly distributed because fragmentation was dominated by formation of the y9 ion with a N-terminal proline although this peptide yielded the more sensitive assays (Table 1) [22]. When depletion with the MARS Hu-6 column was coupled with filter-aided digestion, the dynamic range of the assay was extended with transitions being stable down to 1.28 fmol on-column for Ara h 1 (P43237)^329–34^ and 0.97 fmol on-column for Ara h 1 (P43237)^329–34^ (Fig. 2a). Consequently, the LLOQ values were reduced to 3.85 and 2.75 fmol on-column for each of the peptides, respectively (Table 1).

**Table 1:** Effect of different serum depletion strategies on the LOD and LLOQ of synthetic peanut allergen peptide SIDs in serum. Serum was depleted using either a ProteoPrep^®^ 20 Plasma Immunodepletion Kit (depleted 1) or a MARS Hu-6 (depleted 2). LOD: 3sy|*x*/slope; LLOQ: LOD*3. Instrument LOD and LLOQ are expressed as femtomoles of peptide on-column. *ND*, not determined due to a lack of an adequate calibration curve; *FAD*, filter-aided digestion; *QQQ*, triple quadrupole MRM analysis; *Q-TOF*, quadrupole time-of-flight MRM analysis. The LOD and the LLOQ are expressed in femtomoles on-column. Additional information of the linear regression parameters can be found in ESM Tables S2.1 and S2.2

Peptide	Sample	MS type	LOD	LLOQ	Peptide	Sample		LOD	LLOQ
Ara h 1 (P43237)^329–342^	Serum	QQQ	ND	ND	Ara h 2 (Q6PSU2)^103–115^	Serum	QQQ	ND	ND
	Depleted (1)	QQQ	ND	ND		Depleted (1)	QQQ	ND	ND
	Depleted (2)	QQQ	2.81	8.43		Depleted (2)	QQQ	ND	ND
	Depleted (2) and FAD	QQQ	2.61	7.82		Depleted (2) and FAD	QQQ	ND	ND
	Depleted (2) and FAD	Q-TOF	1.28	3.85		Depleted (2) and FAD	Q-TOF	1.26	3.78
Ara h 1 (P43237)^555–575^	Serum	QQQ	ND	ND	Ara h 2 (Q6PSU2)^147–155^	Serum	QQQ	207.90	ND
	Depleted (1)	QQQ	58.71	176.12		Depleted (1)	QQQ	141.60	ND
	Depleted (2)	QQQ	7.57	22.72		Depleted (2)	QQQ	1.47	4.41
	Depleted (2) and FAD	QQQ	0.92	2.75		Depleted (2) and FAD	QQQ	0.79	2.38
	Depleted (2) and FAD	Q-TOF	Not analysed	Not analysed		Depleted (2) and FAD	Q-TOF	0.60	1.79
Ara h 3 (Q647H4)^25–41^	Serum	QQQ	ND	ND	Ara h 6 (Q647G9)^136–144^	Serum	QQQ	ND	ND
	Depleted (1)	QQQ	91.10	273.31		Depleted (1)	QQQ	ND	ND
	Depleted (2)	QQQ	1.65	4.95		Depleted (2)	QQQ	4.58	13.75
	Depleted (2) and FAD	QQQ	1.61	4.84		Depleted (2) and FAD	QQQ	5.68	17.03
	Depleted (2) and FAD	Q-TOF	Not analysed	Not analysed		Depleted (2) and FAD	Q-TOF	Not analysed	Not analysed
Ara h 3 (Q647H4)^372–384^	Serum	QQQ	ND	ND	Ara h 7 (B4X1D4)^143–151^	Serum	QQQ	ND	ND
	Depleted (1)	QQQ	51.28	153.84		Depleted (1)	QQQ	113.39	ND
	Depleted (2)	QQQ	3.05	9.14		Depleted (2)	QQQ	4.75	14.25
	Depleted (2) and FAD	QQQ	0.95	2.82		Depleted (2) and FAD	QQQ	0.84	2.53
	Depleted (2) and FAD	Q-TOF	0.53	1.60		Depleted (2) and FAD	Q-TOF	Not analysed	Not analysed

#### Ara h 3

**Fig. 3:**
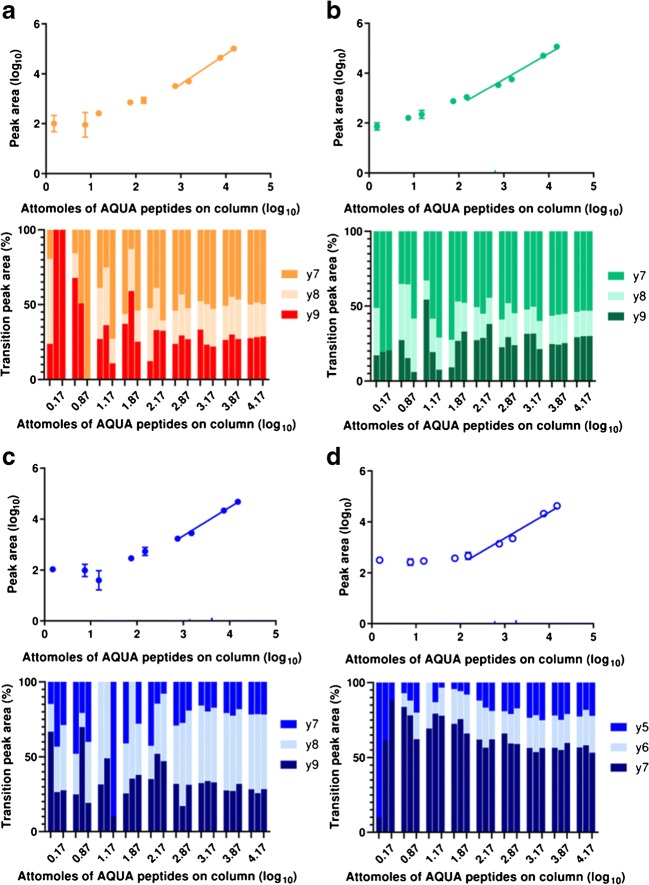
Serial isotopic dilution curves and corresponding transitions for heavy labelled peanut allergen peptide markers in depleted serum analysed using the Q-TOF-MRM method. Peptides were as follows: **a** Ara h 1 (P43237)^329–342^; **b** Arah3 (Q647H4)^372–384^; **c** Ara h 2 (Q6PSU2)^103–115^; **d** Ara h 2 (Q6PSU2)^147–155^. Serum samples were depleted with a MARS-Hu6 column. Calibration curves are created using peptide concentrations from 0 to 15 fmol/μL using samples with qualifying signal-to-noise ratios and ratios of quantifying peak area to total peak area

Again, like the Ara h 1 peptides, neither Ara h 3 (Q647H4)^25–41^ nor Ara h 3 (Q647H4)^372–384^ could be detected in serum although both could be detected in depleted serum (ESM Figs. S2.2 and S2.3). However, serum depleted with the MARS Hu-6 column (depleted 2) gave LOD and LLOQ values which were almost 100-fold lower than when using the spin column depletion method (depleted 1; Table 1 and ESM Figs. S2.2–2.4) and in a similar to those obtained for the Ara h 1 reporter peptides. The most intense transition in both peanut protein extract and peanut protein spiked in the serum for the peptide Ara h 3 (Q647H4)^25–41^ was the y10 ion followed by the y6 and y7 ions. The selective and ready fragmentation of peptides on the N-terminal proline is expected [31, 34]. However, when used for the analysis of peanut in food matrices, utilising the same instrumentation and chromatography as our QQQ, the transitions had a different pattern of intensity with the y6 ion dominating [22] (ESM Fig. S2.6). For Ara h 3 (Q647H4)^372–384^, the y7 ion was the most intense followed by the y8 and y9 ions as observed in peanut flour extracts [16] and transitions proved much more stable than had been the case for analysis of food matrices [22]. The Ara h 3 (Q647H4)^372–384^ achieved a much lower level of detection (Fig. 3b) with stable transitions consistently observed in samples with ≥ 0.53 fmol of peptide on-column. Including filter-aided digestion in the sample workflow had no effect on assay sensitivity for Ara h 3 (Q647H4)^25–41^ but had significantly lowered assay sensitivity for peptide Ara h 3(Q647H4)^372–384^ giving a LOD of 0.95 and LLOQ of 2.82 fmol on-column (Table 1 and Fig. 2).

#### Ara h 2, 6, and 7

None of the peptide reporters for the 2S albumin allergens could be quantified in serum although the Ara h 2 (Q6PSU2)^147–155^ peptide could be detected in serum albeit only at around 200 fmol of peptide on-column (Fig. 1, Table 1, and ESM Figs. S2.2 and S2.3). Depletion improved the LOD and LOQ for all the peptides except Ara h 2 (Q6PSU2)^103–115^, the MARS Hu-6 column treatment again showing the best improvement (Fig. 1, Table 1, and ESM Fig. S2.4). The Ara h 2 (Q6PSU2)^147–155^ peptide reporter could be detected at the lowest level of all the peptides, with a LOQ of 4.4 fmol on-column. The same patterns of transitions were observed for these peptides as were previously observed for analysis of peanut and food matrix extracts [16, 22] (ESM Figs. S2.7 and S2.8). The application of the filter-aided digestion did not improve the performance of either of the Ara h 2 peptide reporters although the LOD and LLOQ achieved for peptides Ara h 2 (Q6PSU2)^147–155^ and Ara h 7 (B4X1D4)^143–151^ were lowered further by around 2–5-fold (Fig. 2).

In stage 3 of the method development, an additional strategy to improve the sensitivity of the method was explored using a Q-TOF instrument. This was done to test whether the enhanced mass accuracy of the Q-TOF could help differentiate the peanut peptide masses from interfering peptide masses from the serum proteins (Fig. 3 and Table 1). For this assessment, a subset of four peptides was used where filter-aided digestion had either slightly improved (Ara h 3(Q647H4)^372–384^ and Ara h 2 (Q6PSU2)^147–155^) or not changed (Ara h 1 (P43237)^329–342^ and Ara h 2 (Q6PSU2)^103–115^) assay sensitivity. The Q-TOF MRM method allowed all the peptides to be reproducibly detected with assay sensitivities of 0.53–1.3 fmol on-column, the patterns of transitions being more reproducible even at the higher concentrations in the SIDs (Table 1, Fig. 3, and ESM Figs. S2.5-S2.7). This is evidenced by the lower CV% and standard deviations observed for the monitored transition ratios at each spike dose level and replicate for the Q-TOF method (Table 1, Figs. 2 and 3, and ESM Tables S2.3 and S2.4). Specifically the method extended the dynamic range of the assay for Ara h 1 (P43237)^329–34^ by around 2-fold to give LOD and LLOQ values of 1.28 and 3.85 fmol on-column, respectively (Fig. 3a and Table 1). The Ara h 2 (Q6PSU2)^147–155^ peptide performed similarly in the QQQ- and Q-TOF MRM methods. There was a notable improvement in the performance of the Ara h 2 (Q6PSU2)^103–115^ peptide with all three transitions reproducibly detected in samples containing the peptide reported at levels as low as 1.26 fmol on-column. The chromatographic separation of the peptides achieved using the HSS T3 Column coupled with the Q-TOF allowed the separation of the Ara h 2(Q6PSU2)^103–115^ and the Ara h 3 (Q647H4)^372–384^ peptides that were co-eluting when the separation was performed using the ionKey (ESM Fig. S2.1). This, together with the greater mass accuracy provided by the TOF analyser, contributed to increasing the sensitivity for these two peptides.

### Determination of peanut in serum: comparison of targeted MS and ELISA

A Tris-HCl buffer extract of raw peanut that was used to spike the serum samples for the spike-recovery experiments was analysed with regard to its allergen composition by SDS PAGE/densitometry and ELISA (ESM Fig. S2.9 and Tables S2.6 and S2.7). This buffer has previously been shown to be more efficient at extracting peanut proteins than another physiologically compatible buffer, phosphate-buffered saline, with an extraction efficiency of ~ 100% [16]. The SDS-PAGE/densitometry and ELISA gave consistent results with Ara h 3 being the most abundant allergen in the extract representing approximately 35–40% of the total extract protein, followed by Ara h 1 which accounted for 15% of the total extract protein. The proportions of both Ara h 1 and Ara h 3 were lower than anticipated which probably results from no reducing agent being used in the preparation of the extract [16]. The estimation of the content of the prolamin allergens, Ara h 2 and Ara h 6, determined by SDS-PAGE and ELISA was also similar with each allergen comprising around 5–10% of the protein extract, although the ELISA appeared to slightly underestimate the Ara h 2 content compared with the SDS-PAGE analysis (ESM Fig. S2.9 and Tables S2.6 and S2.7). The extract was also analysed using the Q-TOF MRM method to develop a set of conversion factors using the heavy labelled peptide standards using an approach previously used for determination of peanut in incurred food samples [22, 35] to allow reporting of the levels of peanut protein in serum (ESM Tables S2.7).

**Fig. 4:**
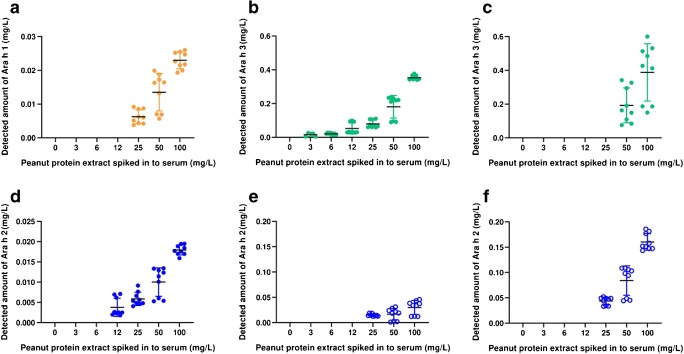
Analysis of serum samples spiked with a total peanut extract using either the QQQ or Q-TOF MRM method. Panels **a**, **b**, **d**, and **e** represent analysis undertaken using the Q-TOF MRM method with panels **c** and **f** representing the QQQ method. Peptide reporters were as follows: **a** Ara h 1 (P43237)^329–342^; **b**, **c** Ara h 3 (Q647H4)^372–384^; **d** Ara h 2 (Q6PSU2)^103–115^; **e**, **f** Ara h 2 (Q6PSU2)^147–155^. Mass of allergen was calculated first by inferring the concentration of reporter peptide using the peak area ratio of the light peptide in the sample to corresponding heavy isotopically labelled peptide standard, then correcting the dilution factors involved in the sample preparation and then multiplying the peptide mass by the mass of the mature protein from which it was derived

**Fig. 5:**
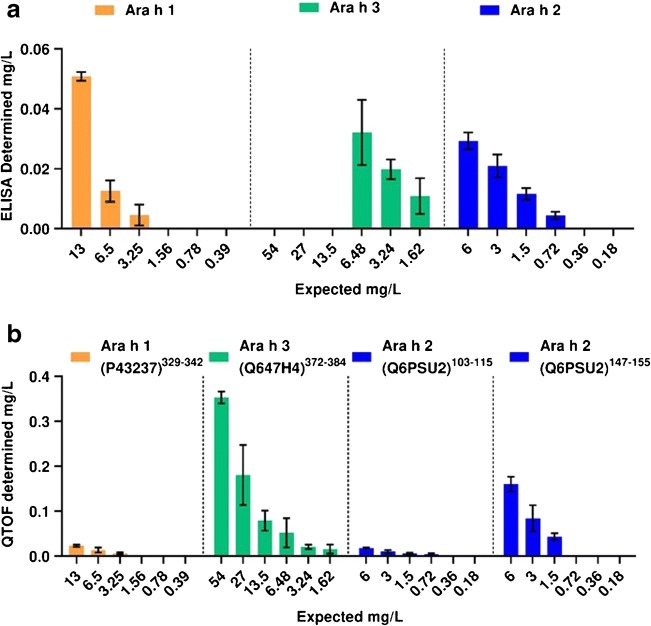
Comparison of peanut allergen determination in spike plasma by ELISA (**a**) and Q-TOF-MRM (**b**). ELISA analysis of the peanut extract was used to calculate the percentage of each allergen in the extract. Calculated values were used to estimate the amount of allergen in each spiked samples assuming no losses during processing. This was compared with the amount of allergen detected in the spiked serum samples using the same ELISA kits (**a**) or the Q-TOF-MRM method (**b**)

Serum samples were spiked with the raw peanut extract and subjected to the sample workflow employing the MARS Hu-6 column for depletion and filter-aided digestion prior to analysis using the QQQ and Q-TOF MRM methods (ESM Fig. S1.1 and Fig. 4). With regard to the QQQ-MRM method neither the Ara h 1 (P43237)^329–342^ nor the Ara h 2 (Q6PSU2)^103–115^ reporter peptides could detect allergen in the peanut-spiked serum samples because of either inconsistent transitions or insufficient peak intensity above the signal-to-noise ratio (Fig. 4a and ESM Table S1.5). The Ara h 3 reporter peptide Ara h 3 (Q647H4)^372–384^ was able to determine the presence of peanut in samples with an initial spike of 50 mg of peanut protein/L. The Ara h 2 (Q6PSU2)^147–155^ peptide could be detected in serum spiked with 25 mg peanut protein/L serum despite Ara h 2 being present at a lower level in the peanut extract (Fig. 4c, f). The results also show much larger variation across biological replicates evident by large error bars, and increased variation in transition ratios. In contrast when analysed using the Q-TOF-MRM method, all four peptides were detected in the spiked serum samples with more consistent transition and, peak area ratios, and providing dose-responses with the amount of spiked-in total peanut protein (Fig. 4a, b, d, e). For example, the peptide reporters Ara h 1 (P43237)^329–342^ and Ara h 2 (Q6PSU2)^147–155^ were both able to quantify peanut protein incurred at a level of 25 mg peanut protein/L of serum, the Ara h 2 (Q6PSU2)^103–115^ peptide performing slightly better and able to determine peanut in the 12-mg peanut protein/L serum sample. The Ara h 3 (Q647H4)^372–384^ peptide performed best, being able to determine peanut down to 3 mg peanut protein/L of serum. The levels of individual allergens in the peanut extract–incurred serum samples were estimated based on the analysis of the extract using the ELISA (ESM Fig. S2.9 and Table S 2.6) and used to compare the ability of the Q-TOF-MRM method and allergen-specific ELISAs to recover the individual allergens (Fig. 5). Recoveries were broadly similar for the Ara h 1, Ara h 3, and Ara h 2 ELISAs and Q-TOF-MRM methods, reporting only ~ 0.6–0.1% of the incurred protein apart from the Ara h 2 (Q6PSU2)^147–155^ peptide which gave better recoveries of ~ 2.5%.

## Conclusions

Serum is a complicated protein-rich matrix comprising a large number of different protein species spanning a wide dynamic range of concentrations from proteins such as serum albumin at milligrammes per litre, to cytokine IL-6 at picogrammes per litre. Of the serum proteins, only ten make up 90% of the serum proteome and include the following: albumin (~ 50%), immunoglobulins (IgGs, IgAs, IgMs), α-2 macroglobulin, α-1 antitrypsin, transferrin, fibrinogen, haptoglobin, and C3-complement factor [36]. The large dynamic range of biological samples, such as serum, can be limiting in a typical MS sample preparation workflows, and an excess of serum peptides in targeted MS samples can lead to ion suppression effects and reduced sensitivity. For this reason, depletion of the most abundant proteins proved necessary in order to allow determination of peanut allergens in serum by mass spectrometry with the MARS Hu-6 depletion column proving the most effective across the entire suite of peanut allergen reporter peptides. However, the LOD and LLOQ values were generally around 10-fold higher than was achieved in the food matrices [22]. One difference in the assay protocols was the filter-aided digestion employed for food analysis, which allowed removal of interfering compounds. Although compounds, such as cocoa phenolics, are not present in serum, the filter has the potential to remove endogenous metabolites. When applied to the preparation of serum samples, it did improve assay sensitivity for several peptide reporters, suggesting that the removal of serum metabolites, which could provide interference effects (e.g. lipids), may also be beneficial for targeted proteomics MS methods for proteins.

The use of a TOF-MRM method with nano-flow chromatography allowed one peptide reporter, Ara h 2 (Q6PSU2)^103–115^, to be analysed much more effectively than proved to be the case with the QQQ-MRM method. The improved performance of this peptide using the Q-TOF methodology may reflect in part differences in the chromatographic separation employed allowing Ara h 2 (Q6PSU2)^103–115^ to be readily resolved from Ara h 3 (Q647H4)^372–384^ (ESM Fig. S2.1). The additional mass accuracy of the Q-TOF may also have enabled better discrimination of the reporter peptide from other peptides within the samples. Additionally, the peptide transitions are more reproducible than those of the QQQ method, providing greater reliability even when not having any effect on the LOD or LLOQ values inferred from the SIDs.

The analysis of serum spiked with peanut protein showed that the Ara h 3 (Q647H4)^372–384^ peptide reporter was able to allow detection of peanut in serum at levels comparable with an ELISA although there are issues over quantification for all allergens using either method. Another factor that requires consideration is that as part of normal immune responses, individuals often have circulating IgG to dietary protein, forming immune complexes that interfere with the analysis of dietary proteins and allergens in serum by ELISA [11]. The MARS Hu-6 depletion column, which proved most effective in this study, will have removed allergens bound in IgG immune complexes. This could account for the low recoveries of peanut allergens from depleted serum samples, although it proved essential to enable the detection of allergens by mass spectrometry. Refinement of sample preparation protocols through inclusion of treatment steps to dissociate such immune complexes prior to depletion for improved recoveries would also allow the detection of peanut allergens in serum at lower levels. Other authors have not addressed fully these issues in the application of analytical test methodology to the analysis of circulating allergens. It may be that test results have all under-estimated the true levels of circulating allergens, which have tended to be in the order of 100’s–1000’s ng/L of allergen [12].

An additional complexity when determining dietary proteins in the circulation is any modification that might occur because of gastrointestinal digestion or changes that result from their uptake into the circulation [37]. Digestion of allergens has the potential to reduce or even abolish antibody binding because the resulting polypeptide fragments are not generally well recognised. Since MS analysis is undertaken at the peptide level, it has the potential to provide a more robust method for confirming uptake of allergens than ELISA, providing peptide targets are contained within digestion fragments [16, 37]. The two best performing reporter peptides, Ara h 3 (Q647H4)^372–38^ and Ara h 2 (Q6PSU2)^147–155^, used in this study both have the potential to address such issues since they are found in allergen fragments which have been identified following simulated digestion [9, 10]. The MS method developed and validated for the analysis of serum has a similar analytical capability with the ELISA and can therefore provide an effective confirmatory method for allergen determination in biological fluids. Development of protocols for disruption of immune complexes will be essential to allow quantification of allergens in serum by both methods in future and allow proper evaluation of modifying factors, such as exercise which may reduce thresholds of reaction [38] and which has been found to enhance allergen uptake in healthy volunteers [39] and individuals with IgE-mediated food allergies [12].

## Electronic supplementary material

ESM 1 (PDF 1896 kb)
