# Supplementary material for: A multiple reaction monitoring method for determining peanut (Arachis hypogea) allergens in serum using quadrupole and time-of-flight mass spectrometry
Source: Anal Bioanal Chem. 2020 Mar 3;412(12):2815–27. doi: 10.1007/s00216-020-02508-9 (PMC7196080; doi:10.1007/s00216-020-02508-9)
Supplement: Supplementary file 1 — (PDF 1896 kb) [file 216_2020_2508_MOESM1_ESM.pdf]

Analytical and Bioanalytical Chemistry

Electronic Supplementary Material

A multiple reaction monitoring method for determining peanut (*Arachis hypogea*) allergens in serum using quadrupole and time-of-flight mass spectrometry

Charlotte M Hands, Lee A Gethings, Rebekah Sayers, E N Clare Mills

Contents

S1. Supplementary Methods 4

Figure S1.1: Method development stages for the detection of peanut allergen in a serum matrices using mass spectrometry. .... **Fehler! Textmarke nicht definiert.**

Table S1.1: MS settings used in MS data aquistion. 5

Table S1.2: Skyline settings 6

S1.1 Quality assurance of mass spectra 6

Table S1.3: MRM transitions and collision energies for heavy and light target peptides generated by Skyline 8

Table S1.4: Peanut allergen characteristics and peptide targets 9

2 Supplementary Results 10

Table S2.1: Linear regression parameters for SIDs derived using data obtained in serum before and after depletion analysed using a triple quadrupole mass spectrometer equipped with an iKey microfluidic device. 11

Table S2.2: Linear regression parameters for SIDs derived using data obtained for depleted serum a Q-TOF mass spectrometer MRM 12

Figure S2.1: Ion chromatograms showing the retention time of each peanut allergen reporter peptide analysed either using (a) a triple quadrupole MRM method using an IonKey microfluidics separation or (b) a Q-TOF MRM method using nanofluidic separation. 12

Table S2.3 Statistical analysis of transition ratios in QQQ-MRM analysis of serum spiked with peanut protein. 13

Table S2.4 statistical analysis of transistion ratios in Q-TOF-MRM analysis of serum spiked with peanut protein. 13

Figure S2.2: Transitions for heavy labelled peanut allergen peptide markers peptides in serum analysed using a triple quadrupole MRM method. 14

Figure S2.3: Transitions for heavy labelled peanut allergen peptide markers peptides in serum depleted using the Proteoprep spin columns analysed using a triple quadrupole MRM method. 15

Figure S2.4: Transitions for heavy labelled peanut allergen peptide markers peptides in serum depleted using the MARS Hu-6 columns analysed using a triple quadrupole MRM method. 16

Figure S2.5: Extracted ion chromatogram of (a, b) Ara h 1(P43237)$^{329-342}$ (VLLEENAGGEQEER) and (c) Ara h 1(P43237)$^{555-577}$ (DLAFPGSGEQVEK used in triple quadrupole (a,c) and Q-TOF MRM (b) experiments showing peptide transitions 17

Figure S2.6: Extracted ion chromatogram of (a, b) Ara h 3 (Q647H4)$^{372-384}$ (SPDIYNPQAGSLK)) and (c) Ara h 3 (Q647H4)$^{25-41}$ (QQPEENACQFQR) used in triple quadrupole (a,c) and Q-TOF MRM (b) experiments showing peptide transitions. 18

Figure S2.7: Extracted ion chromatogram of (a, b) Ara h 2 (Q6PSU2)$^{103-115}$ (CCNELNEFENNQR) and (c, d) Ara h 2 (Q6PSU2)$^{147-155}$(NLPQQCGLR) used in triple quadrupole (a,c) and Q-TOF MRM (b, d) experiments showing peptide transitions. 19

Figure S2.8: Extracted ion chromatogram of (a) Ara h 6 peptide Ara h 6 (Q647G9)$^{136-144}$ and (b) Ara h 7 (B4X1D4)$^{143-151}$ (NLPQNCGFR) used in triple quadrupole MRM experiments showing peptide transitions. 20

Figure S2.9. Characterisation of peanut extract used for serum spiking 21

Table S2.5: Relative quantification of peanut allergens polypeptides in a raw peanut protein extract determined by SDS-PAGE and densitometric analysis. SD- standard deviation; %CV - % coefficient of variation.. 22

Table S2.6: Determination of peanut allergens in a raw peanut protein extract determined by ELISA. SD- standard deviation; %CV - % coefficient of variation... 22

Table S2.7 Conversion factors calculated from MRM analysis of raw peanut extract used for serum spiking. 23

3. Supplementary References.. 24

S1. Supplementary Methods

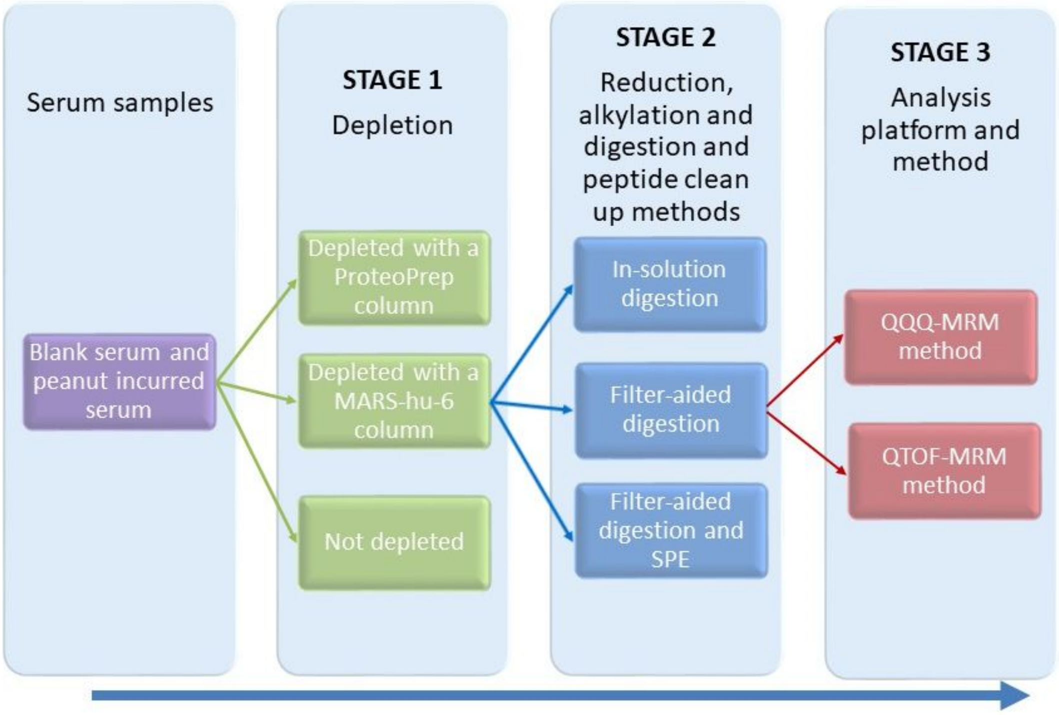

Fig. S1.1 Method development stages for the detection of peanut allergen in a serum matrices using mass spectrometry. In stage one two different methods of depletion were compared, in stage two, three different methods of reduction, alkaylation and digestion were assessed and stage three MS method development was undertaken using two different instrument platforms.

Table S1.1 MS settings used in MS data aquistion

Parameter	Mass Spectrometry Platform	
	**QQQ**	**Q-TOF**
**Polarity**	ESI+	ESI+
**Capillary (kV)**	4.2	3.2000
**Source Temperature (°C)**	100	100
**Sampling Cone**	10.00-190.0	30.0000
**Source Offset**	120.0	30.0000
**Cone Gas Flow (L/Hr)**	35	35.0
**Nanoflow Gas Pressure (Bar)**	0.25	0.2
**Purge Gas Flow (mL/h)**	Off	800.0
**Nebuliser Gas Flow (Bar)**	7.0	6.0
**LM Resolution**	3.2	5.0
**HM Resolution**	14.2	15.0
**Ion Energy**	0.3	0.2

Table S1.2 Skyline settings

A method was created in skyline using the transitions listed in Table S1.2 belowFehler! Verweisquelle konnte nicht gefunden werden.. The MRM .raw data files were imported into Skyline and data visually assessed regarding retention time-matched peak areas corresponding to the total ion intensity for each of the three transition.

**Peptide settings**	
**Enzyme**	Trypsin
**Max missed cleavages**	0
**Time window**	4
**Length range**	8-25
**Modifications**	Carbamidomethyl (C)
**Label**	13C(6)15N(2) (C-term K)<br>13C(6)15N(4) (C-term R)
**Transition settings**	
**Precursor and product ion mass**	Monoisotopic
**Collison energy**	Waters Xevo
**Product ion selection**	Precursor charges (2+), Ion charges (1+), Ions (y)
**Special ions**	N terminal to proline
**Ion match tolerance**	0.5 (*m/z*) with 5 product ions
**Retention time filtering**	Only scans within 5 minutes of MS/MS IDs

S1.1 Quality assurance of mass spectra

Raw files were initially imported into Skyline (1) and the peak picking manually reviewed using retention times and transitions for the peanut allergen peptide reporters (Table S1.1) observed at the highest concentration of labelled target peptides. For the Q-TOF data the peak-area and retention times of Glu-fib peptide for quality assurance of the LC-MS were also visually checked to ensure they were consistent across all samples and the peak picking correct. The data were then exported to excel for further assessment. Spectra where the Glu-fib peptide retention time shifted by ≥1 min from the mean were removed from the analysis.

Data were retained when the intensity of the raw analytical signal was 10 times greater that the noise of the blank serum matrix for the quantifying transition and three times greater for the qualifying transition. Two out of three technical replicates had to meet this criterion for a biological replicate to be included in the analysis. The consistency of transitions was assessed using the minimal-pairs method **where** the relative ratio of the quantifier transition (as described in the Table S1.3) is calculated by dividing its peak area by the total peak area from the same precursor (2).

The reference mean peak area-ratio was calculated using the highest concentration of the labelled AQUA peptides incurred into the serum samples. The samples with ratio measurements falling within +/- 20% of the reference mean peak-area ratio (PAR) were retained. Samples were discarded when 2 out 3 technical replicates did not have a peak-area ratios within +/- 20% of the mean PAR for each relevant peptide. The peak area ratios (PARs) of the heavy-labelled peptide standard to the light peptide in serum samples spiked with peanut extract were calculated based on the total ion intensity (three MRM transitions) using Skyline.

The amount of light peanut peptide reporter in serum was then calculated as follows (2):

$$fentamoles\ of\ light\ peptide = PAR * fentamoles\ of\ heavy\ labelled\ spike$$

S1.2 Factors for conversion of peptide targets to peanut protein

The amount of light peptide reported was used to calculate an amount of allergen per milligram of sample using the molecular weight of the dominant isoform for each protein, assuming molar equivalence between peptides and mature parent protein subunit with the signal peptide removed (Table S1.4) (3, 4). Factors were then calculated for converting from the mass of allergen to mg of total peanut protein using the process flow described by Sayers et al (4) and data from analysis of the allergens in the peanut extract determined by either the triple quadrupole and the QTOF MRM methods (Table S2.7).

Table S1.3 MRM transitions and collision energies for heavy and light target peptides generated by Skyline

Precursor and product ions m/z values are monoisotopic. Quantifier ions are marked using an asterisk, and remaining ions are qualifiers. Quantification was achieved using a total ion counts but the quantifying ion was used to assess ratios of quantifying peak area to total peak area.

Target peptide	y ion	Collision Energy (z)	Un-labelled		Labelled	
			Parent Ion (m/z)	Daughter Ion (m/z)	Parent Ion (m/z)	Daughter Ion (m/z)
Arah1(P43237)[329-342]	y9	28	786.8786	989.4283	791.8828	999.4365
	y8	28	786.8786	875.3854	791.8828	885.3936
	y7*	28	786.8786	804.3482	791.8828	814.3565
Arah1(P43237)[555-577]	y9*	24	688.8383	930.4527	692.8454	938.4669
	y8	24	688.8383	833.3999	692.8454	841.4141
	y7	24	688.8383	776.3785	692.8454	784.3927
Arah3(Q647H4)[103-115]	y10*	27	767.8388	1278.553	772.8429	1288.561
	y7	27	767.8388	923.4152	772.8429	933.4235
	y6	27	767.8388	809.3723	772.8429	819.3806
Arah3(Q647H4)[147-155]	y9	25	695.3541	977.5051	699.3612	985.5193
	y8	25	695.3541	814.4417	699.3612	822.4559
	y7*	25	695.3541	700.3988	699.3612	708.413
Arah2(Q6PSU2)[25-41]	y9	31	863.849	1163.544	868.8532	1173.552
	y8*	31	863.849	1050.46	868.8532	1060.468
	y7	31	863.849	936.417	868.8532	946.4253
Arah2(Q6PSU2)[372-384]	y7*	19	543.2797	858.425	548.2838	868.4333
	y6	19	543.2797	761.3723	548.2838	771.3806
	y5	19	543.2797	633.3137	548.2838	643.322
Arah6(Q647G9)[136-144]	y8	17	489.7191	818.4003	494.7232	828.41
	y7	17	489.7191	703.3733	494.7232	713.38
	y6*	17	489.7191	590.2893	494.7232	600.30
Arah7(B4X1D4)[143-151]	y7*	19	553.264	878.3937	558.2681	888.402
	y6	19	553.264	781.341	558.2681	791.3493
	y5	19	553.264	653.2824	558.2681	663.2907

Table S1.4 Peanut allergen characteristics and peptide targets

The positions of heavy isotopically labelled residues in the synthetic peptide reported are shown in bold (from Sayers et al (3)). The carbamidomethylated cysteine residues are underlined. The molecular masses of peanut allergens were used to convert from peptide to protein mass in development of conversion factors (Table 2.4). The BLAST sequence analysis, performed in November 2019 using UNIPROT BLAST tool, confirmed that these peptides do not show sequence homology with proteins in either the UniProt reference proteome of Homo sapiens or the Viridiplantae database.

**Protein Family**	**Cupin**		**Prolamin**		
**Allergen name**	Ara h 1	Ara h 3	Ara h 2	Ara h 6	Ara h 7
**UniProt ID**	P43237	Q647H4	Q6PSU2	Q647G9	B4X1D4
**Subunit Mr (kDa) (mature protein)**	61.72	59.64	17.99	14.85	17.38
**Peptide Target Sequence (s)**	^{329}VLLEENAGGEQEE**R**^{342}	^{25}QQPEENACQFQ**R**^{41}	^{103}$\underline{CC}$NELNEFENNQ**R**^{115}	^{136}$\underline{C}$DLDVSGG**R**^{144}	^{143}NLPQN$\underline{C}$GF**R**^{151}
	^{555}DLAFPGSGEQVE**K**^{577}	^{372}SPDIYNPQAGSL**K**^{384}	^{147}NLPQQ$\underline{C}$GL**R**^{155}		

2 Supplementary Results

Table S2.1 Linear regression parameters for SIDs derived using data obtained in serum before and after depletion analysed using a triple quadrupole mass spectrometer equipped with an iKey microfluidic device.
Serum was depleted using either a ProteoPrep ® 20 Plasma Immuno-depletion Kit (Depleted 1) or with the MARS Hu-6 column (Depleted 2). FAD – filter aided digestion N/A – not applicable as no linear regression could be calculated due to too few data points.

**Peptide Name**	**Sample Type**											
	**Serum**			**Depleted 1**			**Depleted 2**			**Depleted 2 with FAD**		
	**R^2**	**Line equation**	**sy.x**	**R^2**	**Line equation**	**sy.x**	**R^2**	**Line equation**	**sy.x**	**R^2**	**Line equation**	**sy.x**
Arah1 (P43237)$^{329-342}$	N/A	N/A	N/A	0.9492	Y = 1.088*X + 0.7929	0.1780	0.9892	Y = 0.9842*X + 1.629	0.09478	0.9608	Y = 1.116*X + 2.417	0.2059
Arah1 (P43237)$^{555-577}$	N/A	N/A	N/A	0.9878	Y = 0.9045*X + 2.187	0.07124	0.9861	Y = 0.9802*X + 1.962	0.1070	0.9460	Y = 1.111*X + 2.718	0.2424
Arah3 (Q647H4)$^{25-41}$	N/A	N/A	N/A	0.9565	Y = 0.8348*X + 1.657	0.1259	0.9911	Y = 1.013*X + 1.280	0.1145	0.9106	Y = 1.156*X + 2.078	0.3307
Ara h 3 (Q647H4)$^{372-384}$	N/A	N/A	N/A	0.9935	Y = 0.6684*X + 3.431	0.03812	0.9851	Y = 0.9963*X + 1.920	0.1298	0.9408	Y = 1.206*X + 2.323	0.2762
Arah2 (Q6PSU2)$^{103-115}$	N/A	N/A	N/A	N/A	N/A	N/A	0.9169	Y = 0.8540*X + 0.9693	0.2367	N/A	N/A	N/A
Arah2 (Q6PSU2)$^{147-155}$	0.9225	Y = 0.5302*X + 3.926	0.1236	0.9179	Y = 0.4808*X + 4.504	0.1324	0.9963	Y = 0.9228*X + 2.180	0.05954	0.9493	Y = 1.136*X + 2.776	0.2397
Arah6 (Q647G9)$^{136-144}$	N/A	N/A	N/A	N/A	N/A	N/A	0.9960	Y = 0.9228*X + 2.663	0.2465	0.9444	Y = 1.113*X + 2.226	0.2465
Arah7 (B4XID4)$^{143-151}$	N/A	N/A	N/A	0.8651	Y = 0.7932*X + 2.549	0.2520	0.9767	Y = 0.9007*X + 2.613	0.1470	0.9444	Y = 1.113*X + 2.226	0.2465

Table S2.2 Linear regression parameters for SIDs derived using data obtained for depleted serum a Q-TOF mass spectrometer MRM
Serum was depleted using a MARS Hu-6 column (Depleted 2) together with filter-aided digestion (FAD).

**Peptide Name**	**Depleted 2**		
	**R^2**	**Line equation**	**sy.x**
Arah1(P43237)$^{329-342}$	0.9911	Y = 1.199*X - 0.01026	0.08390
Arah3(Q647H4)$^{372-384}$	0.9761	Y = 1.041*X + 0.6273	0.1501
Arah2(Q6PSU2)$^{103-115}$	0.9899	Y = 1.121*X - 0.01850	0.08348
Arah2(Q6PSU2)$^{147-155}$	0.9723	Y = 1.019*X + 0.3063	0.1585

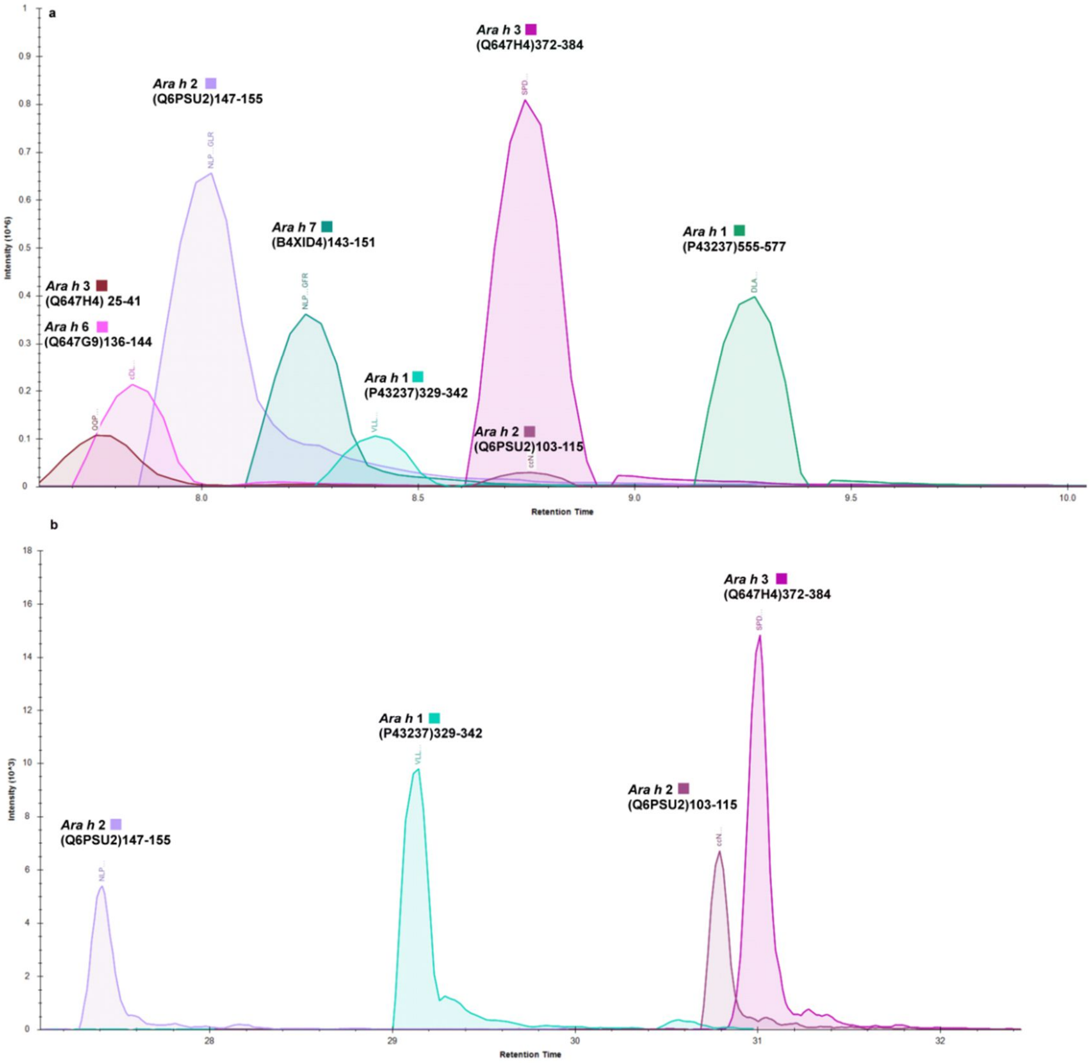

Fig. S2.1 Ion chromatograms showing the retention time of each peanut allergen reporter peptide analysed either using (a) a triple quadrupole MRM method using an IonKey microfluidics separation or (b) a Q-TOF MRM method using nanofluidic separation

Table S2.3 Statistical analysis of transition ratios in QQQ-MRM analysis of serum spiked with peanut protein
SD- standard deviation; %CV - % coefficient of variation

**Protein Family**	**Allergen name**	**Peptide Target Sequence (s)**	**100 ppm**		**50 ppm**		**25 ppm**		**12 ppm**		**6 ppm**		**3 ppm**	
			**SD**	**CV**	**SD**	**CV**	**SD**	**CV**	**SD**	**CV**	**SD**	**CV**	**SD**	**CV**
**Cupin**	Ara h 1	^{329}VLLEENAGGEQEE**R**^{342}	13.80	27.40	10.54	21.65	18.42	33.42	11.47	27.56	12.73	26.09	10.61	18.16
	Ara h 3	^{372}SPDIYNPQAGSL**K**^{384}	6.53	12.86	2.66	5.48	7.72	16.208	5.905	13.069	7.86	16.79	7.31	19.69
**Prolamin**	Ara h 2	^{103}CCNELNEFENNQ**R**^{115}	14.19	26.004	26.87	61.27	13.99	28.601	23.29	34.608	25.17	50.32	26.94	48.31
		^{147}NLPQQCGL**R**^{155}	2.248	3.38	9.02	14.35	3.92	5.78	3.86	5.79	13.75	23.18	11.44	19.76

Table S2.4 statistical analysis of transistion ratios in Q-TOF-MRM analysis of serum spiked with peanut protein

**Protein Family**	**Allergen name**	**Peptide Target Sequence (s)**	**100 ppm**		**50 ppm**		**25 ppm**		**12 ppm**		**6 ppm**		**3 ppm**	
			**SD**	**CV**	**SD**	**CV**	**SD**	**CV**	**SD**	**CV**	**SD**	**CV**	**SD**	**CV**
**Cupin**	Ara h 1	^{329}VLLEENAGGEQEE**R**^{342}	3.69	7.58	4.69	9.18	7.64	15.72	7.27	17.42	13.68	31.56	26.18	60.76
	Ara h 3	^{372}SPDIYNPQAGSL**K**^{384}	1.14	2.11	0.91	1.72	1.6	3.13	2.94	5.51	3.108	5.99	3.25	6.37
**Prolamin**	Ara h 2	^{103}CCNELNEFENNQ**R**^{115}	4.26	8.59	4.67	9.709	5.98	12.49	6.908	14.81	8.76	16.24	9.99	20.87
		^{147}NLPQQCGL**R**^{155}	0.83	1.43	1.04	1.82	2.25	4.16	2.25	4.17	3.208	5.606	3.55	6.42

Fig. S2.2 Transitions for heavy labelled peanut allergen peptide markers peptides in serum analysed using a triple quadrupole MRM method.
a - Ara h 1(P43237)$^{329-342}$ (VLLEENAGGEQEER); b- Ara h 1 (P43237)$^{555-577}$ (DLAFPGSGEQVEK); c - Ara h 3 (Q647H4)$^{25-41}$ (QQPEENACQFQR); d - Ara h 3(Q647H4)$^{372-384}$ (SPDIYNPQAGSLK); e - Ara h 2(Q6PSU2)$^{103-115}$ (CCNELNEFENNQR); f - Ara h 2(Q6PSU2)$^{147-155}$(NLPQQCGLR); g - Ara h 6 peptide Ara h 6 (Q647G9)$^{136-144}$ (CDLDVSGGR); h Ara h 7 (B4X1D4)$^{143-151}$ (NLPQNCGFR)

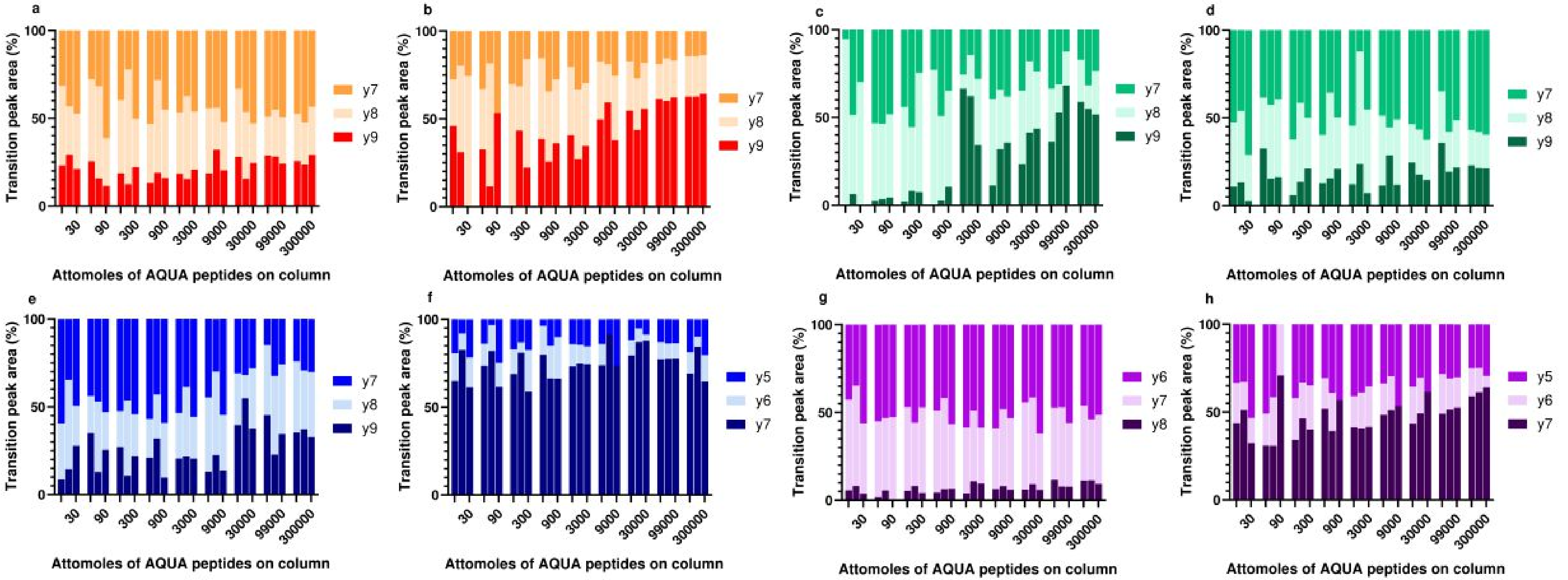

Fig. S2.3 Transitions for heavy labelled peanut allergen peptide markers peptides in serum depleted using the Proteoprep spin columns analysed using a triple quadrupole MRM method

a - Ara h 1(P43237)$^{329\text{-}342}$ (VLLEENAGGEQEER); b- Ara h 1 (P43237)$^{555\text{-}577}$ (DLAFPGSGEQVEK); c - Ara h 3 (Q647H4)$^{25\text{-}41}$ (QQPEENACQFQR); d - Ara h 3(Q647H4)$^{372\text{-}384}$ (SPDIYNPQAGSLK); e - Ara h 2(Q6PSU2)$^{103\text{-}115}$ (CCNELNEFENNQR); f - Ara h 2(Q6PSU2)$^{147\text{-}155}$(NLPQQCGLR); g - Ara h 6 peptide Ara h 6 (Q647G9)$^{136\text{-}144}$ (CDLDVSGGR); h Ara h 7 (B4X1D4)$^{143\text{-}151}$ (NLPQNCGFR).

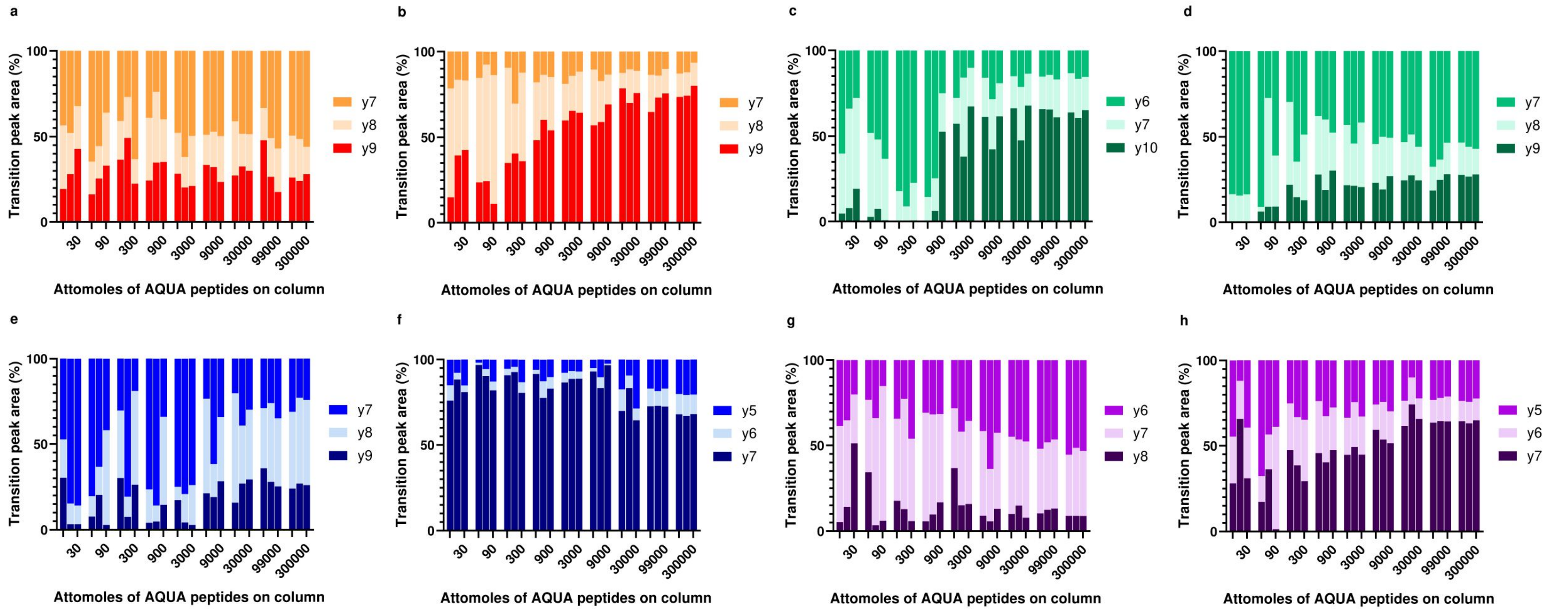

Fig. S2.4 Transitions for heavy labelled peanut allergen peptide markers peptides in serum depleted using the MARS Hu-6 columns analysed using a triple quadrupole MRM method

a - Ara h 1(P43237)$^{329-342}$ (VLLEENAGGEQEER); b- Ara h 1 (P43237)$^{555-577}$ (DLAFPGSGEQVEK); c - Ara h 3 (Q647H4)$^{25-41}$ (QQPEENACQFQR); d - Ara h 3(Q647H4)$^{372-384}$ (SPDIYNPQAGSLK); e - Ara h 2(Q6PSU2)$^{103-115}$ (CCNELNEFENNQR); f - Ara h 2(Q6PSU2)$^{147-155}$(NLPQQCGLR); g - Ara h 6 peptide Ara h 6 (Q647G9)$^{136-144}$ (CDLDVSGGR); h Ara h 7 (B4X1D4)$^{143-151}$ (NLPQNCGFR).

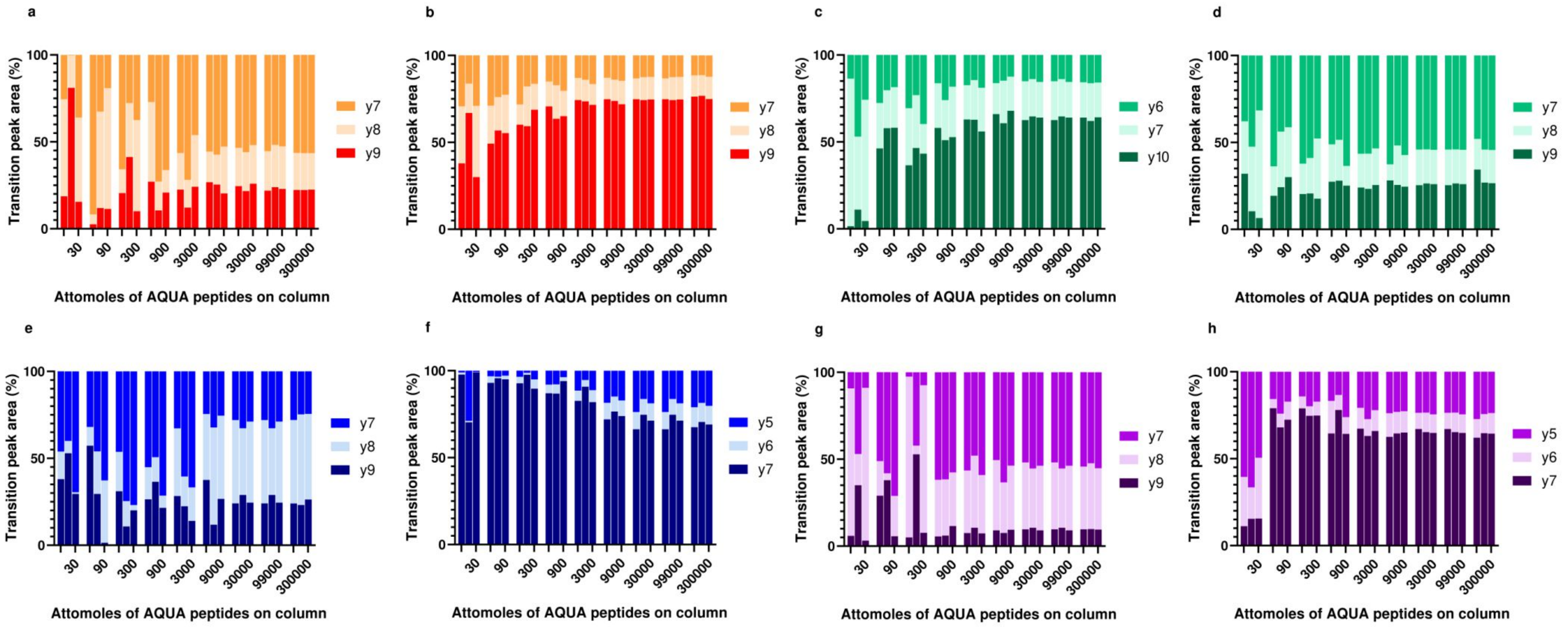

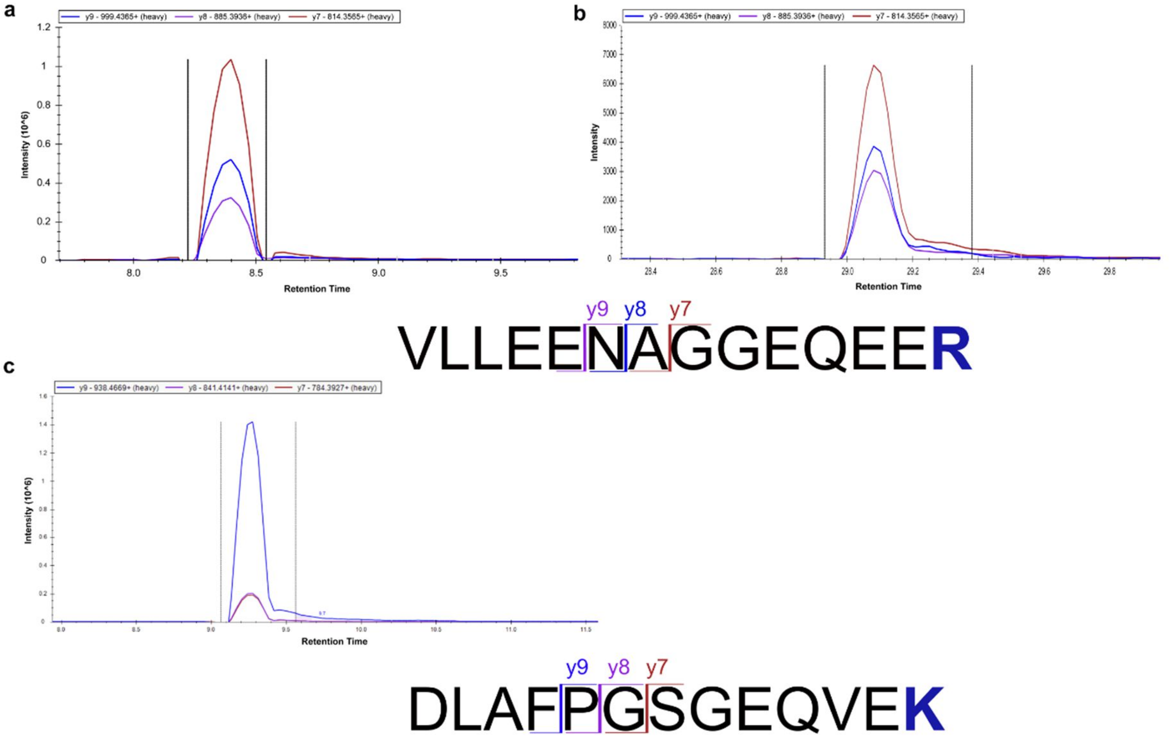

Fig. S2.5 Extracted ion chromatogram of (a, b) Ara h 1(P43237)$^{329\text{-}342}$ (VLLEENAGGEQEER) and (c) Ara h 1(P43237)$^{555\text{-}577}$ (DLAFPGSGEQVEK used in triple quadrupole (a,c) and Q-TOF MRM (b) experiments showing peptide transitions

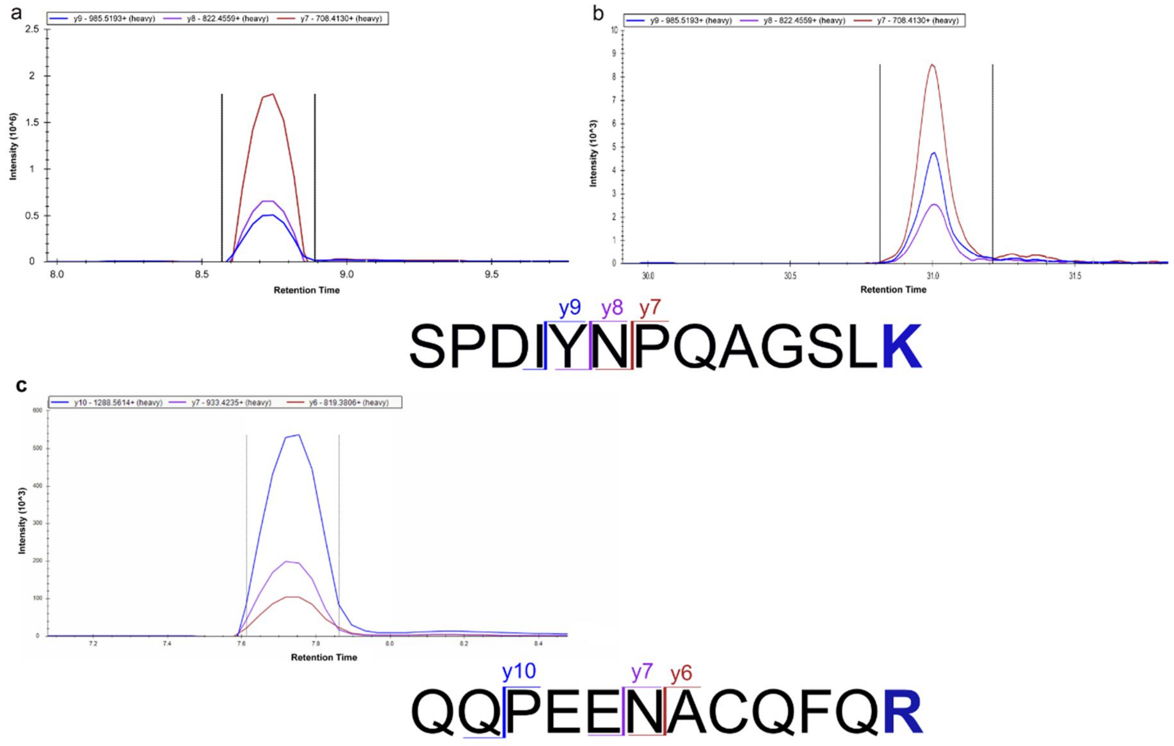

Fig. S2.6 Extracted ion chromatogram of (a, b) Ara h 3 (Q647H4)$^{372\text{-}384}$ (SPDIYNPQAGSLK)) and (c) Ara h 3 (Q647H4)$^{25\text{-}41}$ (QQPEENACQFQR) used in triple quadrupole (a,c) and Q-TOF MRM (b) experiments showing peptide transitions

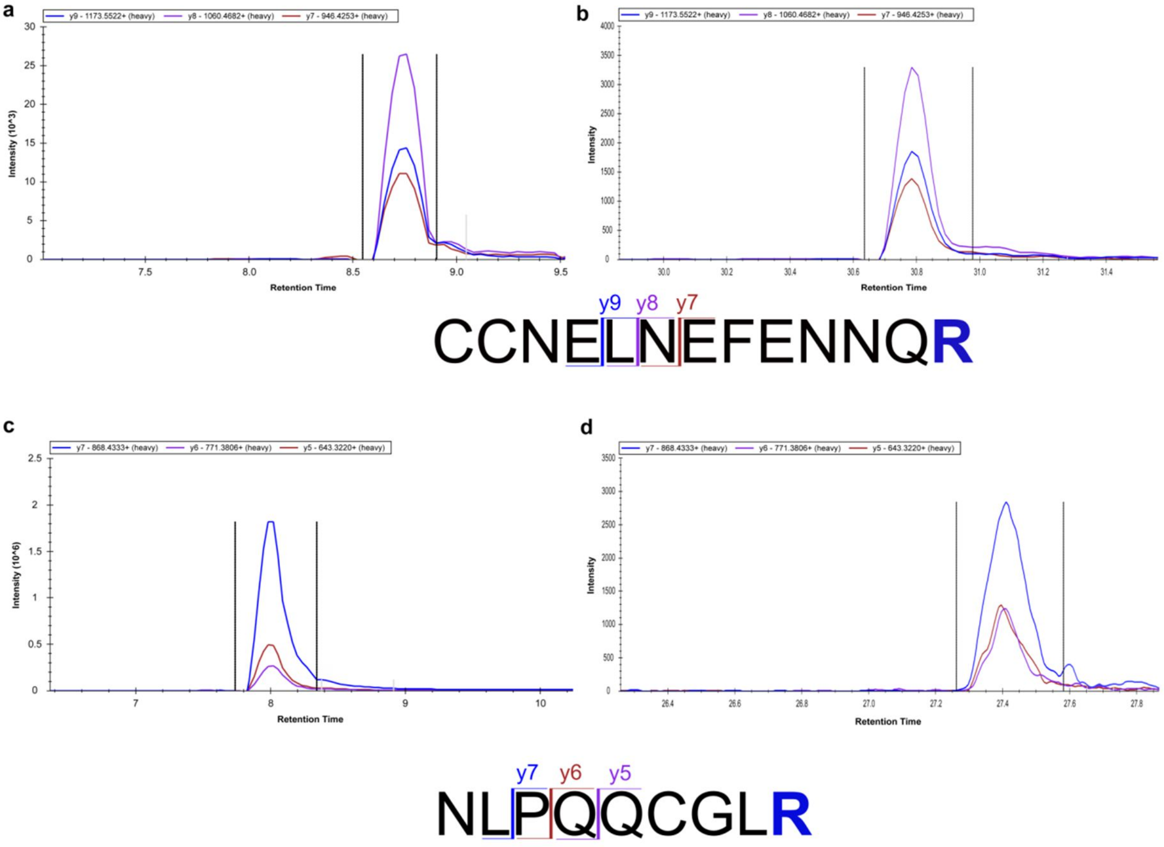

Fig. S2.7 Extracted ion chromatogram of (a, b) Ara h 2 (Q6PSU2)$^{103\text{-}115}$ (CCNELNEFENNQR) and (c, d) Ara h 2 (Q6PSU2)$^{147\text{-}155}$(NLPQQCGLR) used in triple quadrupole (a,c) and Q-TOF MRM (b, d) experiments showing peptide transitions

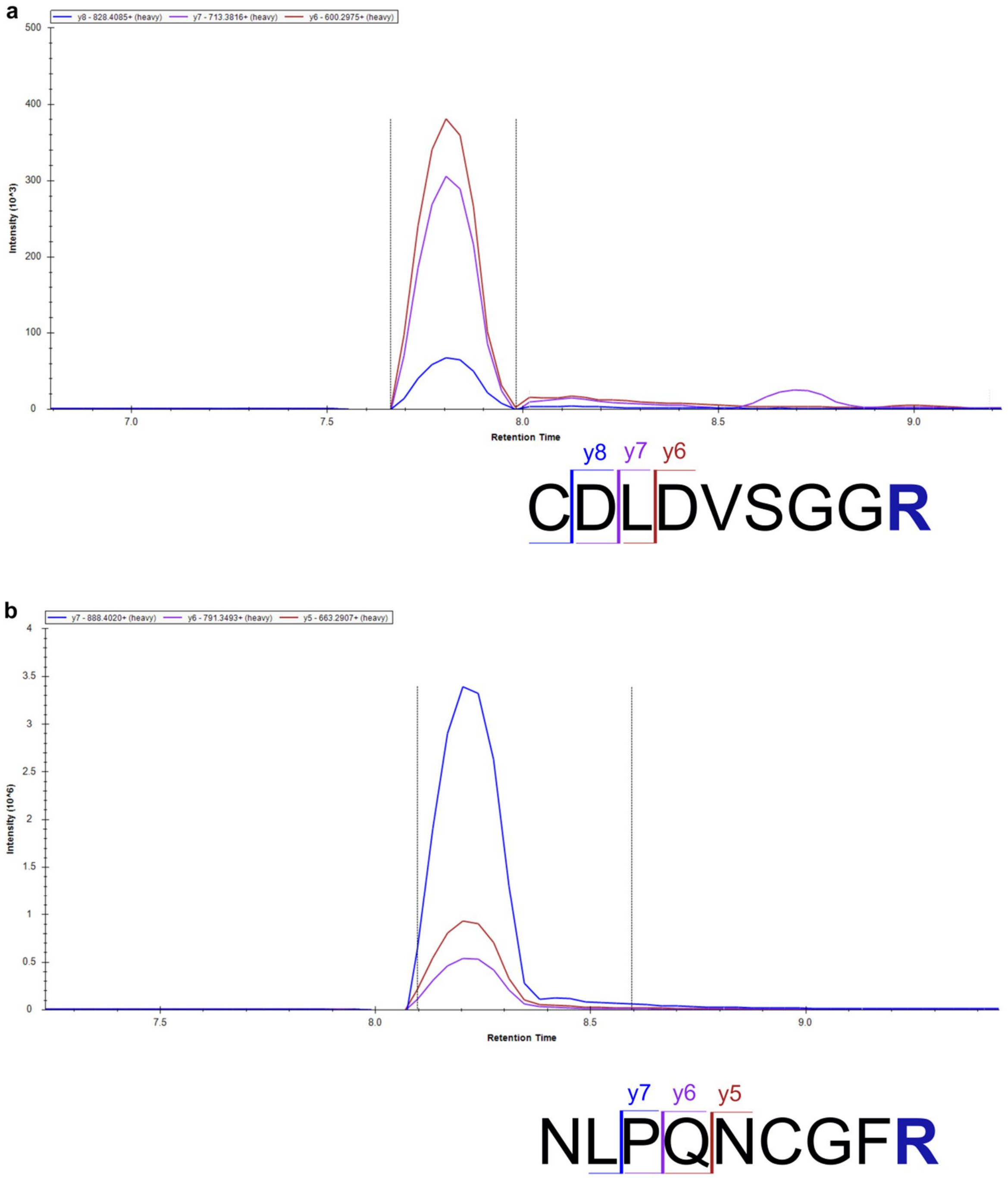

Fig. S2.8 Extracted ion chromatogram of (a) Ara h 6 peptide Ara h 6 (Q647G9)$^{136-144}$ and (b) Ara h 7 (B4X1D4)$^{143-151}$ (NLPQNCGFR) used in triple quadrupole MRM experiments showing peptide transitions

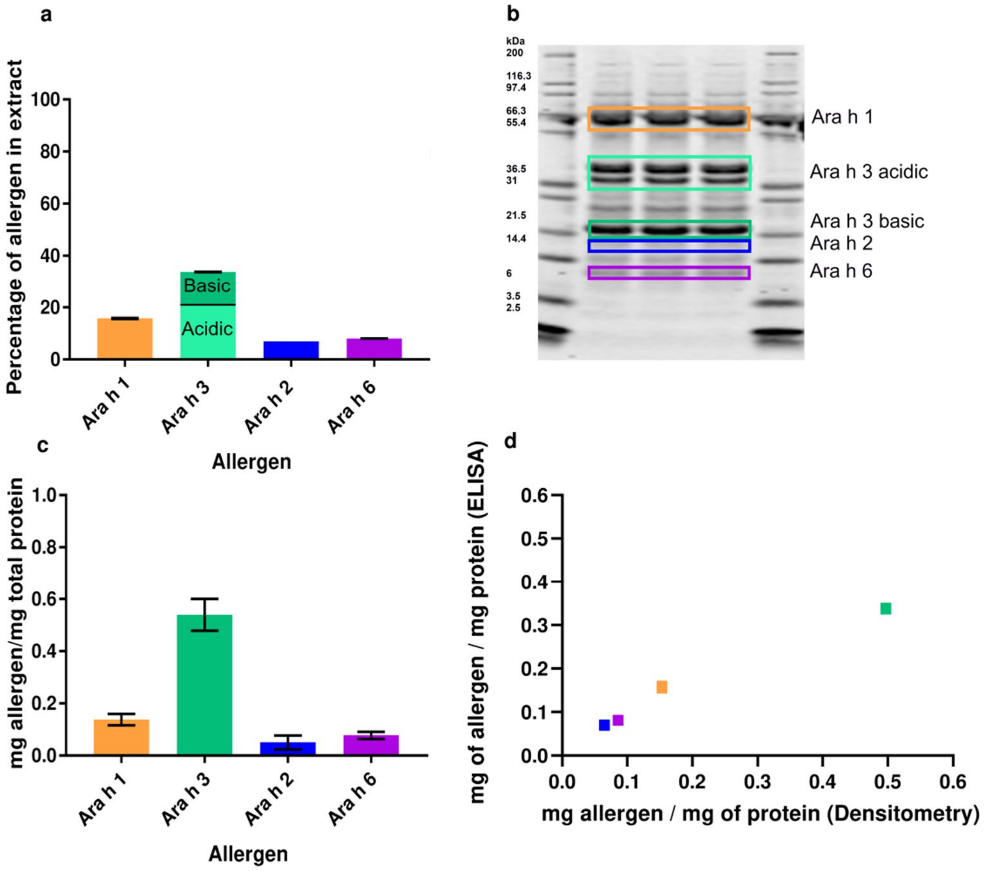

Fig. S2.9 Characterisation of peanut extract used for serum spiking

The raw peanut extract used for serum spiking was characterised either by SDS-PAGE and major allergen bands quantified using densitometry (a,b) or allergen-specific ELISA (c) and the levels of allergens determined by compared (d). Data relating to these figures are presented below in Tables S2.3 and S2.4.

Table S2.5 Relative quantification of peanut allergens polypeptides in a raw peanut protein extract determined by SDS-PAGE and densitometric analysis. SD- standard deviation; %CV - % coefficient of variation

**Peanut allergen**	**% of Total protein staining intensity per track**								
	**Track no**						**Mean**	**SD**	**%CV**
	**1**	**2**	**3**	**4**	**5**	**6**			
Ara h 1	15.48	15.80	15.98	15.48	15.80	15.98	15.75	0.21	1.31
Ara h 3- acidic subunit 1	13.17	13.01	13.00	13.17	13.01	13.00	13.06	0.08	0.60
Ara h 3- acidic subunit 2	7.83	7.87	7.86	7.83	7.87	7.86	7.85	0.02	0.22
Ara h 3- basic subunit	12.85	12.88	12.81	12.85	12.88	12.81	12.85	0.03	0.22
Ara h 2	6.94	7.00	6.95	6.94	7.00	6.95	6.96	0.03	0.38
Ara h 6	8.07	8.05	8.11	8.07	8.05	8.11	8.08	0.02	0.31

Table S2.6 Determination of peanut allergens in a raw peanut protein extract determined by ELISA. SD- standard deviation; %CV - % coefficient of variation

**Peanut allergen**	**mg allergen/mg total peanut protein**	**SD**	**%CV**
Ara h 1	0.13	1.52	11.02
Ara h 3	0.54	4.27	7.92
Ara h 2	0.06	1.06	17.6
Ara h 6	0.077	1.13	14.6

Table S2.7 Conversion factors calculated from MRM analysis of raw peanut extract used for serum spiking
SD- standard deviation; %CV - % coefficient of variation

Conversion factors were calculated for each allergen as described in this supplement section S1.2.

Peptide Target	Triple quadrupole MRM			Q-TOF MRM		
	**Mean µg of allergen per mg of total peanut protein**	**SD**	**%CV**	**Mean µg of allergen per mg of total peanut protein**	**SD**	**%CV**
Ara h 1(P43237)$^{329-342}$	6.648	0.454	6.835	4.908	1.0056	20.486
Ara h 3(Q647H4)$^{372-384}$	20.627	2.547	12.347	9.173	1.834	19.993
Ara h 2(Q6PSU2)$^{103-115}$	12.301	1.644	13.368	2.385	0.153	6.433
Ara h 2(Q6PSU2)$^{147-155}$	24.071	0.267	1.109	7.387	1.123	15.206

3. Supplementary References

1. MacLean B, Tomazela DM, Shulman N, Chambers M, Finney GL, Frewen B, et al. Skyline: an open source document editor for creating and analyzing targeted proteomics experiments. Bioinformatics. 2010;26(7):966-8.
2. Mani DR, Abbatiello SE, Carr SA. Statistical characterization of multiple-reaction monitoring mass spectrometry (MRM-MS) assays for quantitative proteomics. BMC Bioinformatics. 2012;13(16):S9.
3. Sayers RL, Johnson PE, Marsh JT, Barran P, Brown H, Mills EN. The effect of thermal processing on the behaviour of peanut allergen peptide targets used in multiple reaction monitoring mass spectrometry experiments. The Analyst. 2016;141(13):4130-41.
4. Sayers RL, Gethings LA, Lee V, Balasundaram A, Johnson PE, Marsh JA, et al. Microfluidic Separation Coupled to Mass Spectrometry for Quantification of Peanut Allergens in a Complex Food Matrix. Journal of proteome research. 2018;17(1):647-55.
